# Succinct Amyloid and Nonamyloid Patterns in Hexapeptides

**DOI:** 10.1021/acsomega.2c02513

**Published:** 2022-09-27

**Authors:** László Keresztes, Evelin Szögi, Bálint Varga, Viktor Farkas, András Perczel, Vince Grolmusz

**Affiliations:** † PIT Bioinformatics Group, Eötvös University, Budapest H-1117, Hungary; ‡ Uratim Ltd., Budapest H-1118, Hungary; § MTA-ELTE Protein Modeling Research Group, Budapest H-1117, Hungary; ∥ Laboratory of Structural Chemistry and Biology, Eötvös University, Budapest H-1117, Hungary

## Abstract

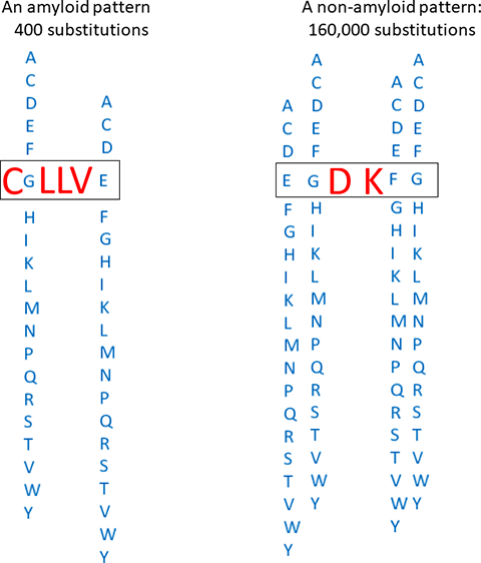

Hexapeptides are widely applied as a model system for studying the amyloid-forming properties of polypeptides, including proteins. Recently, large experimental databases have become publicly available with amyloidogenic labels. Using these data sets for training and testing purposes, one may build artificial intelligence (AI)-based classifiers for predicting the amyloid state of peptides. In our previous work (*Biomolecules***2021**, *11*, 500), we described the Support Vector Machine (SVM)-based Budapest Amyloid Predictor (https://pitgroup.org/bap). Here, we apply the Budapest Amyloid Predictor for discovering numerous amyloidogenic and nonamyloidogenic hexapeptide patterns with accuracy between 80% and 84%, as surprising and succinct novel rules for further understanding the amyloid state of peptides. For example, we have shown that for any independently mutated residue (position marked by “x”), the patterns CxFLWx, FxFLFx, or xxIVIV are predicted to be amyloidogenic, while those of PxDxxx, xxKxEx, and xxPQxx are nonamyloidogenic. We note that each amyloidogenic pattern with two x’s (e.g.,CxFLWx) describes succinctly 20^2^ = 400 hexapeptides, while the nonamyloidogenic patterns comprising four point mutations (e.g.,PxDxxx) give 20^4^ = 160 000 hexapeptides in total. We also examine the restricted substitutions for positions “x” from subclasses of proteinogenic amino acid residues; for example, if “x” is substituted with hydrophobic amino acids, then there exist patterns containing three x’s, like MxVVxx, predicted to be amyloidogenic. If we can choose for the x positions any hydrophobic amino acids, except the “structure breaker” proline, then we get amyloid patterns with five x positions, for example, xxxFxx, each corresponding to 32 768 hexapeptides. To our knowledge, no similar applications of artificial intelligence tools or succinct amyloid patterns were described before the present work.

## Introduction

The amyloid formation of proteins and peptides has gained increasing attention in novel areas of medicine and biology in the last months, including the application of amyloidogenic aggregation cores in viral proteins as new antiviral agents^1^ and the targeting of the lethal transthyretin amyloidosis with human in vivo CRISPR-Cas9-based gene editing with a high success rate.^2^

Amyloids are misfolded proteins^3,4^ with a well-defined and periodic 3D structure, comprising mostly parallel β-sheets.^5,6^ While amyloids are only seldom present in healthy human tissues,^7^ they were reported to be connected with several neurodegenerative diseases,^8^ most importantly with Alzheimer’s disease.

In bioinformatics, numerous amyloid predictors were designed and published in recent years, including APPNN,^9^ Zyggregator,^10^ AGGRESCAN,^11^ and netCSSP;^12^ for a recent review of their performance, we refer to ref (13). These predictors use different machine learning approaches for decision making from training data. In our contribution,^14^ we applied a powerful but transparent machine learning tool, the linear Support Vector Machine (SVM).^15^

For building an SVM, we needed a training data set of *n*-dimensional vectors *y*^1^, *y*^2^, ...*y*^*m*^, each labeled with a bit of either 0 or 1. From the training set, we determine a hyperplane, which, in a certain sense, “optimally” separates the 0- and the 1-labeled vectors in a way that most of the 1-labeled vectors are on one side of the hyperplane and most of the 0-labeled ones are on the other side.

The SVM-predictor now works as follows: If a new vector is situated on the 0-side, then the prediction is “0”, and if it lies on the 1-side, the prediction is “1”.

Recently, we built a Support Vector Machine for the amyloidogenecity prediction of hexapeptides.^14^ The training set was the Waltz data set^16,17^ of experimentally identified 514 amyloidogenic and 901 nonamyloidogenic hexapeptides. By applying the physicochemical property data set of amino acids, AAindex,^18^ we assigned property vectors *y*^1^, *y*^2^, ...*y*^*m*^ to each hexapeptide, and by using this multidimensional representation, we have prepared an SVM, called the Budapest Amyloid Predictor (abbreviated as BAP), described in detail in ref (14) and freely available at https://pitgroup.org/bap.

The accuracy rate of our predictor is 84%, more exactly, ACC = 0.84, TPR = 0.75, TNR = 0.9, PPV = 0.8, NPV = 0.86 (that is, accuracy, true positive ratio, true negative ratio, positive predictive value, and negative predictive value, respectively). We remark that the accuracy of our SVM is better than or on par with that of APPNN,^9^ with a simpler, more transparent structure.

In this Article, we make use of this transparent structure of the Budapest Amyloid Predictor. We present numerous patterns related to amyloidicity, such that each of those patterns grasps hundreds or even tens of thousands of individual hexapeptides and gives predictions of their amyloid-forming properties. For example, we show that for all (independent) substitutions of the 20 amino acids for letter “x”, the hexapeptides CxFLFx, FxFLWx, or xxIVIV are all predicted to be amyloids by the Budapest Amyloid Predictor. Note that each of these patterns describes 20^2^ = 400 different hexapeptides. We also note that no amyloid-forming patterns exist with three x’s for the predictor. All 5531 amyloid-forming hexapeptide patterns with two x’s are listed in Table S1.

We also show several patterns, which, by the Budapest Amyloid Predictor, would not form amyloids. For example, the patterns xxDDxx, xxPxDx, and xxPKxx with any (independently chosen) amino acids for the positions denoted by x are predicted to be nonamyloids by our Budapest Amyloid Predictor. Note that each of these patterns succinctly describes 20^4^ = 160 000 hexapeptides. We add that nonamyloid patterns with five x positions do not exist for our tool at https://pitgroup.org/bap. All of the nonamyloid forming hexapeptide patterns with four *x* positions are listed in Table 2.

We note that these patterns are succinct representations of the predictions of the Budapest Amyloid Predictor (BAP), whose accuracy rate is 84%;^14^ that is, we do not state, for example, that all CxFLFx hexapeptides are amyloids, but we state that all of them are predicted to be amyloids by the BAP tool. The transparent linear structure of the Support Vector Machines makes possible the derivation of these intuitive, useful, and well-applicable patterns from an artificial intelligence (AI) tool, as we clarify in this Article.

We need to add also that today we are living in the era of fast-developing AI methods and tools in numerous fields of science and technology. Most of these tools work as follows.

Suppose the tool needs to compute a value *f*(*y*) from another value *y*. For constructing such an AI tool, the following steps are applied:

- A large set of previously acquired, correct (*y*, *f*(*y*)) pairs are partitioned into two classes: the training set A and the test set B.
- The training set A is applied to construct a tool, which assigns the predicted value of *f*(*y*), denoted by *f*_p_(*y*), to each *y*.
- The test set B is used for evaluating the correctness of the tool: the predicted value, produced by the tool, *f*_p_(*y*), is compared to the correct, previously known *f*(*y*).

The AI tool is deemed “good” if it is correct for a large enough portion of the test values.

In general, however, it is difficult to get insight into the intrinsic decision mechanisms of a typical AI tool; this is especially true for the deep neural networks, which are applied widely today.

In the case of linear Support Vector Machines,^15^ the decision mechanism is much more transparent, and one can exploit a highly correct SVM for gaining unprecedented scientific information in certain cases.^19^ In this Article, we show a novel and original method for gaining site-specific amyloid-forming properties of amino acids in hexapeptides and preparing amyloid-forming and nonamyloid forming patterns for the succinct representation of the SVM prediction results for hundreds (cf., Table S1) or even tens of thousands (cf., Table 2) of hexapeptides at the same time.

## Methods

We have introduced the Budapest Amyloid Predictor Web server^14^ by applying linear Support Vector Machines as the underlying prediction tool,^15^ and the Waltz data set^16,17^ for training and testing purposes. The Waltz data set consists of 1415 hexapeptides, from which 514 peptides are experimentally labeled as “amyloidogenic” and 901 hexapeptides as “nonamyloidogenic”. The Budapest Amyloid Predictor (https://pitgroup.org/bap) was constructed as follows:

(i) Each amino acid from the 20 proteinogenic ones was characterized by a 553-dimensional vector, corresponding to its physicochemical properties published in AAindex.^18^ Therefore, a hexapeptide was represented by a length 6 × 553 = 3318 vector *z*. We note that this highly redundant representation has given somewhat better predictions than more concise ones^14^ and has not caused any difficulties in what follows.

(ii) By applying a quadratic programming algorithm for SVM computation from the SciKit-learn Python library,^20^ we have computed a vector *w* and a scalar *b* such that if the sign of *w*·*z* + *b* is positive, then the prediction is “amyloidogenic”; otherwise, it is “nonamyloidogenic”, with 84% accuracy, for any vector *z*, representing a hexapeptide.

(iii) One can write the dot product *w*·*z*, with  = 553, as
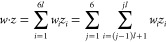
1For any given *j* = 1, 2, ...6, the ’s are determined by the amino acid at position *j* at the hexapeptide. This means that we have only 6 × 20 = 120 sums in eq 1 (for six positions and 20 amino acids), and these 120 values can be precomputed. Table 1 of ref (14) lists these precomputed values. Because we need the same table in the present work, we include it also as Table 1 here.

**Table 1:** Amyloid Effect Matrix, Constructed from the Precomputed Values from Equation 1^a^

	1	2	3	4	5	6
A	–0.26	–0.32	–0.27	–0.14	–0.43	–0.22
R	–0.45	–0.41	–0.46	–0.33	–0.52	–0.35
N	–0.40	–0.34	–0.49	–0.27	–0.46	–0.30
D	–0.49	–0.43	–0.56	–0.41	–0.56	–0.36
C	–0.09	–0.21	0.03	–0.05	–0.17	–0.05
Q	–0.37	–0.30	–0.36	–0.34	–0.48	–0.32
E	–0.51	–0.41	–0.43	–0.30	–0.61	–0.39
G	–0.23	–0.37	–0.46	–0.37	–0.30	–0.33
H	–0.32	–0.26	–0.26	–0.30	–0.35	–0.25
I	–0.06	–0.08	0.26	0.09	–0.06	–0.07
L	–0.10	–0.18	0.02	0.04	–0.22	–0.13
K	–0.39	–0.45	–0.51	–0.35	–0.59	–0.32
M	–0.17	–0.25	–0.02	–0.10	–0.19	–0.18
F	–0.13	–0.11	0.05	–0.03	–0.13	–0.11
P	–0.56	–0.38	–0.56	–0.51	–0.42	–0.45
S	–0.37	–0.35	–0.41	–0.30	–0.48	–0.23
T	–0.34	–0.33	–0.28	–0.23	–0.40	–0.23
W	–0.17	–0.17	–0.09	–0.06	–0.12	–0.16
Y	–0.23	–0.11	–0.13	–0.06	–0.18	–0.15
V	–0.05	–0.14	0.19	0.14	–0.19	0.01

a The rows correspond to the amino acids, while the columns correspond to the positions. The larger numbers show stronger amyloidogenic properties in the given position. Source: ref (14) (Copyright 2021 the authors). In ref (14), by ordering the columns of this table, a position-dependent amyloidogenecity order of amino acids is given in a subsequent table.

(iv) Table 1 makes it possible to decide if a hexapeptide is predicted to be amyloidogenic or not, by “hand”; for example, to decide if IVIVIV is amyloidogenic or not, we need to add up the numbers, corresponding to I in the first, to V in the second, to I in the third, to V in the fourth, to I in the fifth, and to V in the sixth column, that is:
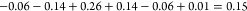
and we need to add to this *w*·*z* value the scalar *b* = 1.083, which equals 1.233, a positive number, so IVIVIV is predicted to be amyloidogenic.

We refer to Table 1 as the Amyloid Effect Matrix.

As we have demonstrated in paragraph (iv) above, one can simply make the prediction of the SVM by using the values solely from this matrix.

From now on, we would like to exploit the Amyloid Effect Matrix for finding succinct descriptions of amyloidogenic and nonamyloidogenic patterns among the 64 million possible hexapeptides.

### Patterns of Amyloidicity

Here, we would like to find very characteristic positions and substitutions, which already ensure us that all of the hexapeptides fitting those patterns are homogeneously either amyloidogenic or nonamyloidogenic. Let us see an example:

#### Example 1

Let us fix the amino acid proline (P) at positions 3 and 4 and leave all four other positions free. Let us consider the pattern
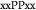


We state that for all (independent) substitutions for x’s, the Budapest Amyloid Predictor (abbreviated as BAP) says that the hexapeptide is not amyloid. Because we have four x’s, the pattern xxPPxx describes exactly 20^4^ = 160 000 hexapeptides, so we state that not one of these 160 000 hexapeptides is predicted to be amyloidogenic.

It is very easy to verify this statement from Table 1. The values corresponding to P’s in the third and in the fourth positions (−0.56 and −0.51) add up to −1.07. Now, even if we take the largest values of columns 1, 2, 5, and 6, that is, −0.05, −0.08, −0.06, and 0.01, respectively, their sum is −1.25, and adding *b* = 1.083 to this value, we would still have a result to be a negative number. That is, even the largest values from columns 1, 2, 5, and 6 could not overweigh the large negative sum of −1.07 of the two consecutive proline residues in positions 3 and 4. This means that all hexapeptides, fitting to the pattern of xxPPxx, are predicted to be nonamyloids by BAP.

#### Example 2

Similarly, one can also find amyloid patterns. For example, we state that all 400 (=20 × 20) hexapeptides, fitting to the pattern FxFLWx, are predicted to be amyloids. One can easily verify this statement from Table 1. The F in position 1 adds −0.13, in position 3 adds 0.05, L in position 4 adds 0.04, and W in position 5 contributes −0.12; their sum is −0.16. Now, if we take the smallest values from columns 2 and 6, that is, −0.45 and −0.45, and add *b* = 1.083 to their sum, we will get −0.16 – 0.45 – 0.45 + 1.083 = 0.023, that is, a positive number, so independently from the choice of the x’s, FxFLWx is predicted to be an amyloid-forming hexapeptide.

### Minimal Patterns

In what follows, we will find all of the minimal patterns of amyloidicity and nonamyloidicity. These minimal patterns are the most concise representations of the amyloid-forming rules of the BAP predictor.

Here, the “minimal” word means that we cannot decrease the number of the fixed amino acids without invalidating the rule. Our goal is to find the patterns with the minimum number of amino acids fixed. From such minimal patterns, one can easily generate valid but nonminimal ones; for example, the xxPPxx pattern is predicted to be nonamyloidogenic for any substitutions of x’s. Therefore, WxPPxx or VIPPxx are also nonamyloid patterns for any substitutions for x, but they are not minimal. It is easy to see by observing Table 1 that neither xxxPxx nor xxPxxx are valid nonamyloid patterns, so xxPPxx is a minimal pattern.

### Finding All Minimal Patterns

Our goal is to find every hexapeptide pattern, both the amyloidogenic and the nonamyloidogenic ones, as predicted by BAP.

Finding these patterns is straightforward using the Amyloid Effect Matrix (Table 1). Suppose that we intend to generate the minimal amyloid indicating patterns. Finding the nonamyloid patterns is a similar procedure.

Verifying whether a pattern is a valid amyloid indicator is easy. We need to generalize the steps done in the examples. We substitute the minimal amyloid effective amino acids on the free positions (denoted by s) and check its score. If the score is already positive, then this least amyloidogenic hexapeptide is already amyloid, and then every other hexapeptide from this space is amyloid too.

Finding the rules for hexapeptides could be done by exhaustive search. Let say we want to find all of the rules with *k* fixed amino acids, where *k* is between 1 and 6. In what follows, we call the core of the rule the number of fixed amino acids (e.g., the core of rule xxPPxx is 2). The positions s will be referred to as free positions.

For finding all of the rules with core *k*, our approach is

(i) generating all of the  index subsets;
(ii) for each index subset, we generate all of the 20^*k*^ rule candidates by assigning all of the possible amino acids to the *k* core positions; and
(iii) verify the validity of the pattern by checking each of them as already described.

We remark that this exact exhaustive search is not fast computationally, but it perfectly works for hexapeptides. The number of verifications is
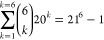
less than 86 million, and its running time is several hours in today’s low-end computers.

The amyloid patterns are listed in Table S1, while the nonamyloid patterns are in Table 2.

### The Case of Restricted Amino Acid Classes

Amino acids are frequently characterized and classified by their chemical properties, like polarity, nonpolarity, hydrophobicity, hydrophilicity, etc. If we want to find patterns of amyloidicity for the free positions, denoted by x, one can choose substitutions only from a given restricted class, and then one can have stronger, more specific patterns than in the general case, when x can be substituted by any of the 20 amino acids.

Finding those patterns in the restricted classes can be done analogously to the general case. The minimum values of Table 1 from the given class need to be considered.

### Statistical Analysis

We refer to the work^14^ for the statistical accuracy estimations of the Budapest Amyloid Predictor. There we have shown that the predictor has ACC = 0.84, TPR = 0.75, TNR = 0.9, PPV = 0.8, and NPV = 0.86 (that is, accuracy, true positive ratio, true negative ratio, positive predictive value, and negative predictive value, respectively). Figure 1 of ref (14) also gives the ROC (receiver operating characteristics) curve of the tool, with the AUC (area under curve) value as 0.89.

## Results and Discussion

**Figure 1:**
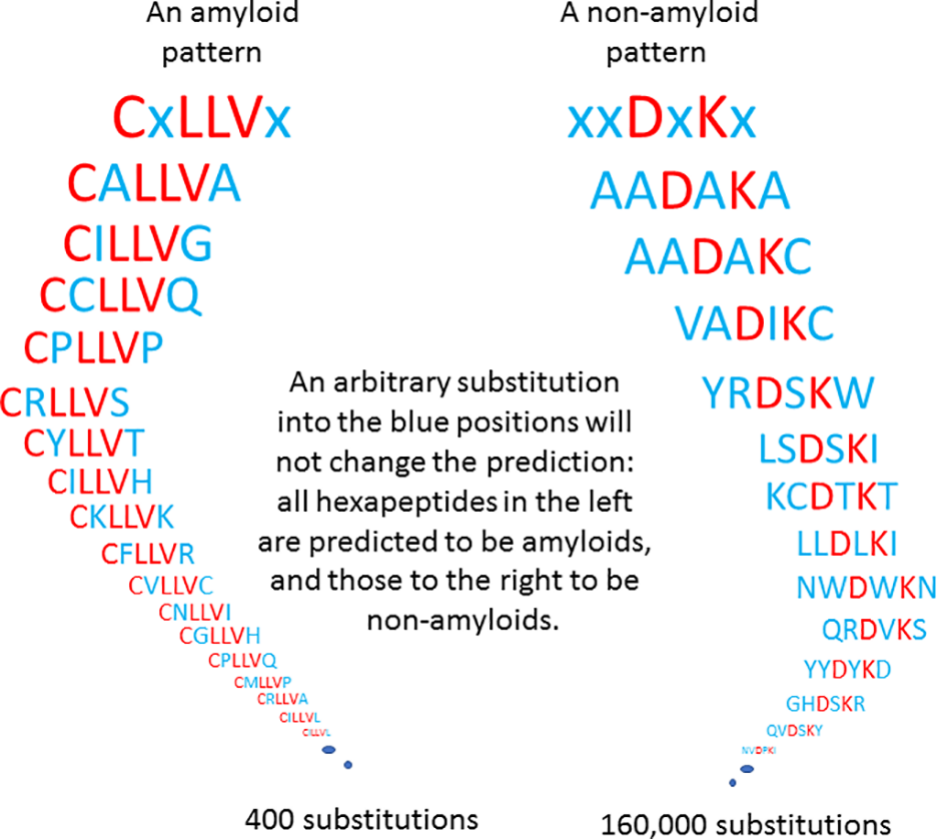
Examples of amyloid and nonamyloid patterns.

Figure 1 visualizes the substitutions into a nonamyloid and an amyloid pattern.

We have successfully identified all 5531 minimal amyloid patterns (Table S1) using BAP. For example, xxIYCI, IFIYxx, and CxVVxC are amyloid patterns from Table S1.

We have found that almost all amyloidogenic hexapeptide patterns contain valine (V) and/or isoleucine (I) residues, both of branched and hydrophobic side chains. Among the 5531 patterns identified (Table S1), only 16 patterns are free of both V and I: CxFLWx, CxFLFx, CxCLWx, CxCLFx, CxLLWx, CxLLFx, LxFLWx, LxFLFx, LxCLWx, LxCLFx, LxLLWx, LxLLFx, FxFLWx, FxFLFx, FxCLWx, and CxFLCx.

Furthermore, leucine, L, the third branched and hydrophobic side chain, is represented one or more times in the above listed 16 patterns. In conclusion, V, I, and L residues make hexapeptides intrinsically amyloidogenic.

We remark that no amyloid pattern with three free positions (i.e., x) exists, by the results of the exhaustive search.

Table 2 lists the 24 nonamyloid patterns, each with four free positions. No other nonamyloid patterns exist with four free positions, and no nonamyloid pattern exists with five free positions by the results of the exhaustive search, described in the Methods.

**Table 2:** List of All Nonamyloid Patterns with Four Free Positions^a^

PxPxxx	PxDxxx	xxPPxx	xxPDxx	xxPGxx	xxPKxx
xxPQxx	xxDPxx	xxDDxx	xxDGxx	xxDKxx	xxDQxx
xxKPxx	xxKDxx	xxNPxx	xxGPxx	xxRPxx	xxPxEx
xxPxKx	xxPxDx	xxDxEx	xxDxKx	xxDxDx	xxKxEx

a It contains 24 patterns. Note that each pattern describes 20^4^ = 160 000 hexapeptides succinctly, all of which are predicted to be nonamyloids by the Budapest Amyloid Predictor.^14^ From the 24 patterns, only nine do not contain proline in a fixed position.

### Results for Amino Acid Subsets

In this subsection, we find amyloid patterns when the x positions can be substituted only by the members of some specific amino acid classes. The amino acid classes we examine are small nonpolar, hydrophobic, and polar amino acids, as classified by ref (21), and listed in the second column of Table 3.

**Table 3:** Amino Acid Subsets Examined^a^

class name	class elements listing	no. of free positions	no. of patterns
small nonpolar	GAST	3	411
hydrophobic	CVLIMPFYW	3	43
polar	DENQHKR	3	4
hydrophobic-{P}	CVLIMFYW	5	38
amino acids-{P}	QFYESNCDMLIAHGWRKVT	3	4

a The classification of the residues in the first three rows is as in ref (21). The last two rows correspond to the classes where we left out proline, a well-known structure-breaker from the hydrophobic set or from all of the amino acids. The third column shows the number of free positions we get in the special substitutions, and the fourth column shows the number of patterns found for these special substitutions for “x”.

When the substitutions to the free positions, denoted by x, can be done only from special subsets, listed in Table 3, we can get amyloid rules with three free positions, in contrast with the unrestricted case, when our rules have two free positions (Table S1).

When x is allowed to be substituted from the small nonpolar set, then 411 patterns can be found with three free positions, for example, VIIxxx, IIIxxx, VxIVxx, VxIIxx, VxILxx, VxIFxx, VxICxx, VxIWxx, VxVVxx, and VxVIxx. All of the existing 411 patterns are listed in Table S2. No such pattern exists with four free positions.

If x is chosen from the hydrophobic set, then 43 patterns exist with three free positions, listed in Table 4. No such pattern exists with four free positions, by the results of the exhaustive search.

**Table 4:** List of All 43 Amyloidogenic Patterns with Three Free Positions When x Is Hydrophobic, Chosen from CVLIMPFYW^a^

VxIVxx	VxIIxx	VxILxx	VxIFxx	VxVVxx	VxVIxx	VxVLxx	IxIVxx
IxIIxx	IxILxx	IxVVxx	IxVIxx	CxIVxx	CxIIxx	CxILxx	CxVVxx
CxVIxx	LxIVxx	LxIIxx	LxILxx	LxVVxx	LxVIxx	FxIVxx	FxIIxx
FxVVxx	MxIVxx	MxIIxx	MxVVxx	WxIVxx	WxIIxx	GxIVxx	YxIVxx
xxIVIx	xxIVxV	xxIVxC	xxIVxI	xxIVxF	xxIIxV	xxIIxC	xxILxV
xxVVxV	xxVVxC	xxVIxV					

a Each pattern describes 9^3^ = 729 hexapeptides.

When x is chosen from polar amino acids, then the only four patterns with three free positions are xxIVIx, xxIVWx, xxIIIx, and xxVVIx.

We note that no pattern exists in these three cases without V and I amino acids; that is, all of the patterns in these three restricted substitutions contain either valine or isoleucine in fixed positions.

If proline is not allowed to be substituted for any x, but otherwise the remaining 19 amino acids can be chosen for the x positions, then we have exactly four amyloid patterns with three x positions: xxIVIx, xxIVWx, xxIIIx, and xxVVIx; note that without the restriction to proline, no amyloid pattern exists with three free positions.

These four patterns are exactly the same as in the case of polar residue substitutions, but the set of hexapeptides they represent differs: in the case of polar substitutions, each of the four patterns represent 7^3^ = 343 hexapeptides, while for the nonproline substitutions, 19^3^ = 6859 hexamers.

If x could be chosen from hydrophobic amino acids, except proline, the “structure breaker”, then we have the “largest” patterns of amyloidicity: 38 patterns exist with just one fixed position, listed in Table 5. Note that each of those patterns describes 8^5^ = 32 768 hexapeptides, such that all of them are predicted to be amyloidogenic.

**Table 5:** List of All 38 Amyloidogenic Patterns with Five Free Positions When x Is Hydrophobic, but Cannot Be Proline, Chosen from CVLIMFYW^a^

Vxxxxx	Ixxxxx	Cxxxxx	Lxxxxx	Fxxxxx	Mxxxxx	Wxxxxx	xIxxxx
xFxxxx	xYxxxx	xVxxxx	xWxxxx	xLxxxx	xCxxxx	xxIxxx	xxVxxx
xxFxxx	xxCxxx	xxLxxx	xxMxxx	xxWxxx	xxxVxx	xxxIxx	xxxLxx
xxxFxx	xxxCxx	xxxWxx	xxxYxx	xxxxIx	xxxxWx	xxxxFx	xxxxCx
xxxxYx	xxxxxV	xxxxxC	xxxxxI	xxxxxF	xxxxxL		

a Each pattern describes 8^5^ = 32 768 hexapeptides.

## Conclusions

Here, we established the patterns of amyloidicity and nonamyloidicity in the case of hexapeptides, based on a Support Vector Machine-based predictor, available at https://pitgroup.org/bap. Because there are 20^6^, that is, 64 million hexapeptides, formed from the 20 proteinogenic amino acids, it is worthwhile to show succinct patterns of both amyloid-forming and nonforming hexapeptides, based on the BAP predictor. First, in the literature, we have introduced hexapeptide patterns with free-to-choose positions, denoted by “x”, describing hundreds, or even tens of thousands of hexapeptides with the same predicted amyloidogenecity, each with only six characters. In Table S1, we list 5531 amyloid patterns (e.g., CxLLVx), where for the positions, denoted by “x”, we can substitute any of the 20 amino acids, and the resulting hexapeptide will be predicted as “amyloidogenic” by BAP. Note that each of the patterns in Table S1 describes 400 hexapeptides. Similarly, we have found succinct representations of the BAP-predicted nonamyloidogenic hexapeptides (Table 2), each with four free positions. Therefore, each entry of Table 2 represents 20^4^ = 160 000 hexapeptides. We have also examined restricted substitutions for the x positions, like small nonpolar, or hydrophobic or polar amino acids, and described succinct patterns for those hexamers in Tables 3, 4, 5, and S2.

To our knowledge, no machine learning tool was used before to derive succinct chemical knowledge through simple patterns for deep structural properties.

## Notes

The Budapest Amyloid Predictor webserver is available freely at https://pitgroup.org/bap. The hexapeptide patterns identified in this work are enclosed in the text or in the Supporting Information.

## Supporting Information Available

Table S1 lists all of the existing minimal patterns of amyloid-forming hexapeptides, computed from the SVM model of the Budapest Amyloid Predictor; Table S2 lists all of the existing minimal patterns of amyloid-forming hexapeptides, computed from the SVM model of the Budapest Amyloid Predictor, when the substitutions for the positions, denoted by “x”, are allowed by small, nonpolar residues, G, A, S, and T (PDF)

## Dedication

Dedicated to the memory of Imre G. Csizmadia.
