# Supplementary material for: Succinct Amyloid and Nonamyloid Patterns in Hexapeptides
Source: ACS Omega. 2022 Sep 27;7(40):35532–7. doi: 10.1021/acsomega.2c02513 (PMC9558248; doi:10.1021/acsomega.2c02513)
Supplement: Supplementary file 1 — ao2c02513_si_001.pdf [file ao2c02513_si_001.pdf]

Supporting Material for the Article: Succinct Amyloid and Non-Amyloid Patterns in Hexapeptides

László Keresztes[a,**], Evelin Szögi[a,**], Bálint Varga[a], Viktor Farkas[c], András Perczel[c,d], Vince Grolmusz[a,b,*]

[a] *PIT Bioinformatics Group, Eötvös University, H-1117 Budapest, Hungary*
[b] *Uratim Ltd., H-1118 Budapest, Hungary*
[c] *MTA-ELTE Protein Modeling Research Group, H-1117 Budapest, Hungary*
[d] *Laboratory of Structural Chemistry and Biology, Eötvös University, H-1117, Budapest, Hungary*

Table S1: Amyloid patterns in hexapeptides

In the table we list all the existing minimal patterns of amyloid-forming hexapeptides, computed from the SVM model of the Budapest Amyloid Predictor. Each pattern in the Table contains two free "x" positions, where any amino acids can be substituted, and the resulting hexapeptide is predicted to be amyloidogenic. Since the two free positions can be substituted $20 \times 20 = 400$ ways, each of the 5531 patterns in the Table describes 400 hexapeptides succinctly.

0	1	2	3	4	5	6	7	8	9
VIIVxx	VIIIxx	VIILxx	VIIFxx	VIICxx	VIIWxx	VIIYxx	VIIMxx	VIIAxx	VIVVxx
VIVIxx	VIVLxx	VIVFxx	VIVCxx	VIVWxx	VIVYxx	VIFVxx	VIFIxx	VICVxx	VICIxx
VILVxx	VILIxx	VIMVxx	VFIVxx	VFIIxx	VFILxx	VFIFxx	VFICxx	VFIWxx	VFIYxx
VFIMxx	VFVVxx	VFVIxx	VFVLxx	VFVFxx	VFVCxx	VFFVxx	VFFIxx	VFCVxx	VFLVxx
VYIVxx	VYIIxx	VYILxx	VYIFxx	VYICxx	VYIWxx	VYIYxx	VYIMxx	VYVVxx	VYVIxx
VYVLxx	VYVFxx	VYVCxx	VYFVxx	VYFIxx	VYCVxx	VYLVxx	VVIVxx	VVIIxx	VVILxx
VVIFxx	VVICxx	VVIWxx	VVIYxx	VVVVxx	VVVIxx	VVVLxx	VVVFxx	VVFVxx	VVCVxx
VVLVxx	VWIVxx	VWIIxx	VWILxx	VWIFxx	VWICxx	VWIWxx	VWIYxx	VWVVxx	VWVIxx
VWVLxx	VLIVxx	VLIIxx	VLILxx	VLIFxx	VLICxx	VLVVxx	VLVIxx	VLVLxx	VCIVxx
VCIIxx	VCILxx	VCVVxx	VCVIxx	VCVLxx	VMIVxx	VMIIxx	VMILxx	VMVVxx	VMVIxx
VHIVxx	VHIIxx	VHILxx	VHVVxx	VHVIxx	VQIVxx	VQIIxx	VQVVxx	VAIVxx	VAIIxx
VTIVxx	VTIIxx	VNIVxx	VSIVxx	VGIVxx	IIIVxx	IIIIxx	IIILxx	IIIFxx	IIICxx
IIIWxx	IIIYxx	IIIMxx	IIIAxx	IIVVxx	IIVIxx	IIVLxx	IIVFxx	IIVCxx	IIVWxx
IIVYxx	IIFVxx	IIFIxx	IICVxx	IICIxx	IILVxx	IILIxx	IIMVxx	IFIVxx	IFIIxx
IFILxx	IFIFxx	IFICxx	IFIWxx	IFIYxx	IFIMxx	IFVVxx	IFVIxx	IFVLxx	IFVFxx
IFFVxx	IFCVxx	IFLVxx	IYIVxx	IYIIxx	IYILxx	IYIFxx	IYICxx	IYIWxx	IYIYxx
IYIMxx	IYVVxx	IYVIxx	IYVLxx	IYVFxx	IYFVxx	IYCVxx	IYLVxx	IVIVxx	IVIIxx
IVILxx	IVIFxx	IVICxx	IVIWxx	IVIYxx	IVVVxx	IVVIxx	IVVLxx	IVFVxx	IVCVxx
IWIVxx	IWIIxx	IWILxx	IWIFxx	IWICxx	IWVVxx	IWVIxx	IWVLxx	ILIVxx	ILIIxx
ILILxx	ILIFxx	ILVVxx	ILVIxx	ILVLxx	ICIVxx	ICIIxx	ICILxx	ICVVxx	ICVIxx
IMIVxx	IMIIxx	IMILxx	IMVVxx	IHIVxx	IHIIxx	IHILxx	IHVVxx	IQIVxx	IQIIxx
IQVVxx	IAIVxx	ITIVxx	INIVxx	ISIVxx	CIIVxx	CIIIxx	CIILxx	CIIFxx	CIICxx

*Corresponding author
**Joint first authors

CIIWxx	CIIYxx	CIIMxx	CIVVxx	CIVIxx	CIVLxx	CIVFxx	CIVCxx	CIFVxx	CIFIxx
CICVxx	CILVxx	CFIVxx	CFIIxx	CFILxx	CFIFxx	CFICxx	CFIWxx	CFIYxx	CFVVxx
CFVIxx	CFVLxx	CFFVxx	CYIVxx	CYIIxx	CYILxx	CYIFxx	CYICxx	CYIWxx	CYIYxx
CYVVxx	CYVIxx	CYVLxx	CYFVxx	CVIVxx	CVIIxx	CVILxx	CVIFxx	CVICxx	CVVVxx
CVVIxx	CVVLxx	CWIVxx	CWIIxx	CWILxx	CWVVxx	CWVIxx	CLIVxx	CLIIxx	CLILxx
CLVVxx	CLVIxx	CCIVxx	CCIIxx	CCILxx	CCVVxx	CCVIxx	CMIVxx	CMIIxx	CMVVxx
CHIVxx	CHIIxx	CHVVxx	CQIVxx	CAIVxx	CTIVxx	LIIVxx	LIIIxx	LIILxx	LIIFxx
LIICxx	LIIWxx	LIIYxx	LIIMxx	LIVVxx	LIVIxx	LIVLxx	LIVFxx	LIFVxx	LICVxx
LILVxx	LFIVxx	LFIIxx	LFILxx	LFIFxx	LFICxx	LFIWxx	LFIYxx	LFVVxx	LFVIxx
LFVLxx	LFFVxx	LYIVxx	LYIIxx	LYILxx	LYIFxx	LYICxx	LYIWxx	LYIYxx	LYVVxx
LYVIxx	LYVLxx	LYFVxx	LVIVxx	LVIIxx	LVILxx	LVIFxx	LVICxx	LVVVxx	LVVIxx
LVVLxx	LWIVxx	LWIIxx	LWILxx	LWVVxx	LWVIxx	LLIVxx	LLIIxx	LLILxx	LLVVxx
LLVIxx	LCIVxx	LCIIxx	LCILxx	LCVVxx	LCVIxx	LMIVxx	LMIIxx	LMVVxx	LHIVxx
LHIIxx	LHVVxx	LQIVxx	LAIVxx	LTIVxx	FIIVxx	FIIIxx	FIILxx	FIIFxx	FIICxx
FIIWxx	FIIYxx	FIVVxx	FIVIxx	FIVLxx	FIFVxx	FFIVxx	FFIIxx	FFILxx	FFIFxx
FFVVxx	FFVIxx	FFVLxx	FYIVxx	FYIIxx	FYILxx	FYIFxx	FYVVxx	FYVIxx	FYVLxx
FVIVxx	FVIIxx	FVILxx	FVVVxx	FVVIxx	FWIVxx	FWIIxx	FWILxx	FWVVxx	FWVIxx
FLIVxx	FLIIxx	FLILxx	FLVVxx	FLVIxx	FCIVxx	FCIIxx	FCVVxx	FMIVxx	FHIVxx
MIIVxx	MIIIxx	MIILxx	MIIFxx	MIVVxx	MIVIxx	MIVLxx	MFIVxx	MFIIxx	MFILxx
MFVVxx	MFVIxx	MYIVxx	MYIIxx	MYILxx	MYVVxx	MYVIxx	MVIVxx	MVIIxx	MVILxx
MVVVxx	MVVIxx	MWIVxx	MWIIxx	MWVVxx	MLIVxx	MLIIxx	MLVVxx	MCIVxx	MCIIxx
MMIVxx	MHIVxx	WIIVxx	WIIIxx	WIILxx	WIIFxx	WIVVxx	WIVIxx	WIVLxx	WFIVxx
WFIIxx	WFILxx	WFVVxx	WFVIxx	WYIVxx	WYIIxx	WYILxx	WYVVxx	WYVIxx	WVIVxx
WVIIxx	WVILxx	WVVVxx	WVVIxx	WWIVxx	WWIIxx	WWVVxx	WLIVxx	WLIIxx	WLVVxx
WCIVxx	WCIIxx	WMIVxx	WHIVxx	GIIVxx	GIIIxx	GIILxx	GIVVxx	GIVIxx	GFIVxx
GFIIxx	GFVVxx	GYIVxx	GYIIxx	GYVVxx	GVIVxx	GVIIxx	GWIVxx	GLIVxx	YIIVxx
YIIIxx	YIILxx	YIVVxx	YIVIxx	YFIVxx	YFIIxx	YFVVxx	YYIVxx	YYIIxx	YYVVxx
YVIVxx	YVIIxx	YWIVxx	YLIVxx	AIIVxx	AIIIxx	AIVVxx	AFIVxx	AFIIxx	AYIVxx
AYIIxx	AVIVxx	HIIVxx	TIIVxx	VIIxIx	VIIxWx	VIIxFx	VIIxCx	VIIxYx	VIIxMx
VIIxVx	VIIxLx	VIVxIx	VIVxWx	VIVxFx	VIVxCx	VIVxYx	VIVxMx	VIVxVx	VFIxIx
VFIxWx	VFIxFx	VFIxCx	VFIxYx	VFIxMx	VFIxVx	VFIxLx	VFVxIx	VFVxWx	VFVxFx
VYIxIx	VYIxWx	VYIxFx	VYIxCx	VYIxYx	VYIxMx	VYIxVx	VYIxLx	VYVxIx	VYVxWx
VYVxFx	VVIxIx	VVIxWx	VVIxFx	VVIxCx	VVIxYx	VVIxMx	VVIxVx	VVVxIx	VVVxWx
VWIxIx	VWIxWx	VWIxFx	VWVxIx	VLIxIx	VLIxWx	VLIxFx	VLVxIx	VCIxIx	VCIxWx
VCVxIx	VMIxIx	VHIxIx	IIIxIx	IIIxWx	IIIxFx	IIIxCx	IIIxYx	IIIxMx	IIIxVx
IIIxLx	IIVxIx	IIVxWx	IIVxFx	IIVxCx	IIVxYx	IFIxIx	IFIxWx	IFIxFx	IFIxCx
IFIxYx	IFIxMx	IFIxVx	IFVxIx	IFVxWx	IFVxFx	IYIxIx	IYIxWx	IYIxFx	IYIxCx
IYIxYx	IYIxMx	IYIxVx	IYVxIx	IYVxWx	IYVxFx	IVIxIx	IVIxWx	IVIxFx	IVIxCx
IVIxYx	IVVxIx	IVVxWx	IWIxIx	IWIxWx	IWIxFx	IWVxIx	ILIxIx	ILIxWx	ILIxFx
ILVxIx	ICIxIx	ICIxWx	IMIxIx	IHIxIx	CIIxIx	CIIxWx	CIIxFx	CIIxCx	CIIxYx
CIIxMx	CIIxVx	CIVxIx	CIVxWx	CIVxFx	CFIxIx	CFIxWx	CFIxFx	CFIxCx	CFIxYx
CFVxIx	CFVxWx	CYIxIx	CYIxWx	CYIxFx	CYIxCx	CYIxYx	CYVxIx	CYVxWx	CVIxIx
CVIxWx	CVIxFx	CVVxIx	CWIxIx	CWIxWx	CLIxIx	CLIxWx	CCIxIx	LIIxIx	LIIxWx
LIIxFx	LIIxCx	LIIxYx	LIIxMx	LIIxVx	LIVxIx	LIVxWx	LIVxFx	LFIxIx	LFIxWx
LFIxFx	LFIxCx	LFVxIx	LYIxIx	LYIxWx	LYIxFx	LYIxCx	LYVxIx	LVIxIx	LVIxWx
LVIxFx	LVVxIx	LWIxIx	LLIxIx	LCIxIx	FIIxIx	FIIxWx	FIIxFx	FIIxCx	FIIxYx
FIVxIx	FIVxWx	FFIxIx	FFIxWx	FFIxFx	FFVxIx	FYIxIx	FYIxWx	FYIxFx	FYVxIx
FVIxIx	FVIxWx	FWIxIx	FLIxIx	MIIxIx	MIIxWx	MIIxFx	MIVxIx	MFIxIx	MYIxIx
MVIxIx	WIIxIx	WIIxWx	WIIxFx	WIVxIx	WFIxIx	WYIxIx	WVIxIx	GIIxIx	YIIxIx
VIIxxV	VIIxxC	VIIxxI	VIIxxF	VIVxxV	VFIxxV	VFIxxC	VFVxxV	VYIxxV	VYIxxC
VYVxxV	VVIxxV	VWIxxV	VLIxxV	IIIxxV	IIIxxC	IIIxxI	IIVxxV	IFIxxV	IFIxxC
IYIxxV	IYIxxC	IVIxxV	IWIxxV	ILIxxV	CIIxxV	CIIxxC	CIVxxV	CFIxxV	CYIxxV
CVIxxV	LIIxxV	LIIxxC	LFIxxV	LYIxxV	LVIxxV	FIIxxV	VIxVIx	IIxVIx	VxIVIx
VxIVWx	VxIVFx	VxIVCx	VxIVYx	VxIVMx	VxIVVx	VxIVLx	VxIVGx	VxIVHx	VxIVTx
VxIVPx	VxIVAx	VxIVNx	VxIVQx	VxIVSx	VxIVRx	VxIIIx	VxIIWx	VxIIFx	VxIICx
VxIIYx	VxIIMx	VxIIVx	VxIILx	VxIIGx	VxIIHx	VxIITx	VxIIPx	VxIIAx	VxIINx
VxIIQx	VxILIx	VxILWx	VxILFx	VxILCx	VxILYx	VxILMx	VxILVx	VxILLx	VxILGx
VxILHx	VxILTx	VxILPx	VxIFIx	VxIFWx	VxIFFx	VxIFCx	VxIFYx	VxIFMx	VxIFVx
VxIFLx	VxIFGx	VxIFHx	VxICIx	VxICWx	VxICFx	VxICCx	VxICYx	VxICMx	VxICVx
VxICLx	VxICGx	VxIWIx	VxIWWx	VxIWFx	VxIWCx	VxIWYx	VxIWMx	VxIWVx	VxIWLx
VxIWGx	VxIYIx	VxIYWx	VxIYFx	VxIYCx	VxIYYx	VxIYMx	VxIYVx	VxIYLx	VxIYGx
VxIMIx	VxIMWx	VxIMFx	VxIMCx	VxIMYx	VxIMMx	VxIMVx	VxIMLx	VxIAIx	VxIAWx
VxIAFx	VxIACx	VxIAYx	VxIAMx	VxIAVx	VxIALx	VxITIx	VxITWx	VxITFx	VxINIx
VxINWx	VxIHIx	VxIEIx	VxISIx	VxVVIx	VxVVWx	VxVVFx	VxVVCx	VxVVYx	VxVVMx
VxVVVx	VxVVLx	VxVVGx	VxVVHx	VxVVTx	VxVVPx	VxVVAx	VxVVNx	VxVIIx	VxVIWx
VxVIFx	VxVICx	VxVIYx	VxVIMx	VxVIVx	VxVILx	VxVIGx	VxVIHx	VxVITx	VxVLIx
VxVLWx	VxVLFx	VxVLCx	VxVLYx	VxVLMx	VxVLVx	VxVLLx	VxVLGx	VxVLHx	VxVFIx
VxVFWx	VxVFFx	VxVFCx	VxVFYx	VxVFMx	VxVFVx	VxVFLx	VxVCIx	VxVCWx	VxVCFx

VxVCCx	VxVCYx	VxVCMx	VxVCVx	VxVCLx	VxVWIx	VxVWWx	VxVWFx	VxVWCx	VxVWYx
VxVWMx	VxVWVx	VxVWLx	VxVYIx	VxVYWx	VxVYFx	VxVYCx	VxVYYx	VxVYMx	VxVYVx
VxVYLx	VxVMIx	VxVMWx	VxVMFx	VxVMCx	VxVMYx	VxVMMx	VxVMVx	VxVMLx	VxVAIx
VxVAWx	VxVAFx	VxVACx	VxVAYx	VxVTIx	VxFVIx	VxFVWx	VxFVFx	VxFVCx	VxFVYx
VxFVMx	VxFVVx	VxFVLx	VxFVGx	VxFIIx	VxFIWx	VxFIFx	VxFICx	VxFIYx	VxFIMx
VxFIVx	VxFILx	VxFLIx	VxFLWx	VxFLFx	VxFLCx	VxFLYx	VxFLMx	VxFLVx	VxFFIx
VxFFWx	VxFFFx	VxFCIx	VxFCWx	VxFWIx	VxFWWx	VxFYIx	VxFYWx	VxFMIx	VxCVIx
VxCVWx	VxCVFx	VxCVCx	VxCVYx	VxCVMx	VxCVVx	VxCVLx	VxCVGx	VxCIIx	VxCIWx
VxCIFx	VxCICx	VxCIYx	VxCIMx	VxCIVx	VxCILx	VxCLIx	VxCLWx	VxCLFx	VxCLCx
VxCLYx	VxCLMx	VxCLVx	VxCFIx	VxCFWx	VxCCIx	VxCCWx	VxCWIx	VxCYIx	VxLVIx
VxLVWx	VxLVFx	VxLVCx	VxLVYx	VxLVMx	VxLVVx	VxLVLx	VxLIIx	VxLIWx	VxLIFx
VxLICx	VxLIYx	VxLIMx	VxLIVx	VxLILx	VxLLIx	VxLLWx	VxLLFx	VxLLCx	VxLLYx
VxLFIx	VxLFWx	VxLCIx	VxLWIx	VxLYIx	VxMVIx	VxMVWx	VxMVFx	VxMVCx	VxMVYx
VxMVMx	VxMVVx	VxMVLx	VxMIIx	VxMIWx	VxMIFx	VxMICx	VxMIYx	VxMIMx	VxMIVx
VxMLIx	VxMLWx	VxMLFx	VxMFIx	VxWVIx	VxWVWx	VxWVFx	VxWVCx	VxWVYx	VxWIIx
VxWIWx	VxWLIx	VxYVIx	VxYVWx	VxYVFx	VxYIIx	IxIVIx	IxIVWx	IxIVFx	IxIVCx
IxIVYx	IxIVMx	IxIVVx	IxIVLx	IxIVGx	IxIVHx	IxIVTx	IxIVPx	IxIVAx	IxIVNx
IxIVQx	IxIVSx	IxIIIx	IxIIWx	IxIIFx	IxIICx	IxIIYx	IxIIMx	IxIIVx	IxIILx
IxIIGx	IxIIHx	IxIITx	IxIIPx	IxIIAx	IxIINx	IxILIx	IxILWx	IxILFx	IxILCx
IxILYx	IxILMx	IxILVx	IxILLx	IxILGx	IxILHx	IxILTx	IxIFIx	IxIFWx	IxIFFx
IxIFCx	IxIFYx	IxIFMx	IxIFVx	IxIFLx	IxIFGx	IxICIx	IxICWx	IxICFx	IxICCx
IxICYx	IxICMx	IxICVx	IxICLx	IxICGx	IxIWIx	IxIWWx	IxIWFx	IxIWCx	IxIWYx
IxIWMx	IxIWVx	IxIWLx	IxIWGx	IxIYIx	IxIYWx	IxIYFx	IxIYCx	IxIYYx	IxIYMx
IxIYVx	IxIYLx	IxIYGx	IxIMIx	IxIMWx	IxIMFx	IxIMCx	IxIMYx	IxIMMx	IxIMVx
IxIMLx	IxIAIx	IxIAWx	IxIAFx	IxIACx	IxIAYx	IxIAMx	IxIAVx	IxIALx	IxITIx
IxITWx	IxITFx	IxINIx	IxIHIx	IxIEIx	IxISIx	IxVVIx	IxVVWx	IxVVFx	IxVVCx
IxVVYx	IxVVMx	IxVVVx	IxVVLx	IxVVGx	IxVVHx	IxVVTx	IxVVPx	IxVVAx	IxVIIx
IxVIWx	IxVIFx	IxVICx	IxVIYx	IxVIMx	IxVIVx	IxVILx	IxVIGx	IxVIHx	IxVITx
IxVLIx	IxVLWx	IxVLFx	IxVLCx	IxVLYx	IxVLMx	IxVLVx	IxVLLx	IxVLGx	IxVLHx
IxVFIx	IxVFWx	IxVFFx	IxVFCx	IxVFYx	IxVFMx	IxVFVx	IxVFLx	IxVCIx	IxVCWx
IxVCFx	IxVCCx	IxVCYx	IxVCMx	IxVCVx	IxVCLx	IxVWIx	IxVWWx	IxVWFx	IxVWCx
IxVWYx	IxVWMx	IxVWVx	IxVWLx	IxVYIx	IxVYWx	IxVYFx	IxVYCx	IxVYYx	IxVYMx
IxVYVx	IxVYLx	IxVMIx	IxVMWx	IxVMFx	IxVMCx	IxVMYx	IxVMMx	IxVMVx	IxVAIx
IxVAWx	IxVAFx	IxVTIx	IxFVIx	IxFVWx	IxFVFx	IxFVCx	IxFVYx	IxFVMx	IxFVVx
IxFVLx	IxFVGx	IxFIIx	IxFIWx	IxFIFx	IxFICx	IxFIYx	IxFIMx	IxFIVx	IxFILx
IxFLIx	IxFLWx	IxFLFx	IxFLCx	IxFLYx	IxFLMx	IxFLVx	IxFFIx	IxFFWx	IxFCIx
IxFCWx	IxFWIx	IxFYIx	IxFMIx	IxCVIx	IxCVWx	IxCVFx	IxCVCx	IxCVYx	IxCVMx
IxCVVx	IxCVLx	IxCIIx	IxCIWx	IxCIFx	IxCICx	IxCIYx	IxCIMx	IxCIVx	IxCILx
IxCLIx	IxCLWx	IxCLFx	IxCLCx	IxCLYx	IxCFIx	IxCFWx	IxCCIx	IxCWIx	IxCYIx
IxLVIx	IxLVWx	IxLVFx	IxLVCx	IxLVYx	IxLVMx	IxLVVx	IxLVLx	IxLIIx	IxLIWx
IxLIFx	IxLICx	IxLIYx	IxLIMx	IxLIVx	IxLILx	IxLLIx	IxLLWx	IxLLFx	IxLLCx
IxLFIx	IxLCIx	IxLWIx	IxLYIx	IxMVIx	IxMVWx	IxMVFx	IxMVCx	IxMVYx	IxMVMx
IxMVVx	IxMVLx	IxMIIx	IxMIWx	IxMIFx	IxMICx	IxMIYx	IxMLIx	IxMLWx	IxWVIx
IxWVWx	IxWVFx	IxWIIx	IxWIWx	IxYVIx	IxYVWx	IxYIIx	CxIVIx	CxIVWx	CxIVFx
CxIVCx	CxIVYx	CxIVMx	CxIVVx	CxIVLx	CxIVGx	CxIVHx	CxIVTx	CxIVPx	CxIVAx
CxIVNx	CxIVQx	CxIVSx	CxIIIx	CxIIWx	CxIIFx	CxIICx	CxIIYx	CxIIMx	CxIIVx
CxIILx	CxIIGx	CxIIHx	CxIITx	CxIIPx	CxIIAx	CxILIx	CxILWx	CxILFx	CxILCx
CxILYx	CxILMx	CxILVx	CxILLx	CxILGx	CxILHx	CxIFIx	CxIFWx	CxIFFx	CxIFCx
CxIFYx	CxIFMx	CxIFVx	CxIFLx	CxIFGx	CxICIx	CxICWx	CxICFx	CxICCx	CxICYx
CxICMx	CxICVx	CxICLx	CxICGx	CxIWIx	CxIWWx	CxIWFx	CxIWCx	CxIWYx	CxIWMx
CxIWVx	CxIWLx	CxIYIx	CxIYWx	CxIYFx	CxIYCx	CxIYYx	CxIYMx	CxIYVx	CxIYLx
CxIMIx	CxIMWx	CxIMFx	CxIMCx	CxIMYx	CxIMMx	CxIMVx	CxIMLx	CxIAIx	CxIAWx
CxIAFx	CxIACx	CxIAYx	CxIAMx	CxIAVx	CxITIx	CxINIx	CxVVIx	CxVVWx	CxVVFx
CxVVCx	CxVVYx	CxVVMx	CxVVVx	CxVVLx	CxVVGx	CxVVHx	CxVVTx	CxVVPx	CxVIIx
CxVIWx	CxVIFx	CxVICx	CxVIYx	CxVIMx	CxVIVx	CxVILx	CxVIGx	CxVIHx	CxVLIx
CxVLWx	CxVLFx	CxVLCx	CxVLYx	CxVLMx	CxVLVx	CxVLLx	CxVLGx	CxVFIx	CxVFWx
CxVFFx	CxVFCx	CxVFYx	CxVFMx	CxVFVx	CxVFLx	CxVCIx	CxVCWx	CxVCFx	CxVCCx
CxVCYx	CxVCMx	CxVCVx	CxVCLx	CxVWIx	CxVWWx	CxVWFx	CxVWCx	CxVWYx	CxVWMx
CxVWVx	CxVWLx	CxVYIx	CxVYWx	CxVYFx	CxVYCx	CxVYYx	CxVYMx	CxVYVx	CxVMIx
CxVMWx	CxVMFx	CxVMCx	CxVMYx	CxVAIx	CxVAWx	CxVAFx	CxFVIx	CxFVWx	CxFVFx
CxFVCx	CxFVYx	CxFVMx	CxFVVx	CxFVLx	CxFIIx	CxFIWx	CxFIFx	CxFICx	CxFIYx
CxFIMx	CxFIVx	CxFILx	CxFLIx	CxFLWx	CxFLFx	CxFLCx	CxFFIx	CxFCIx	CxFWIx
CxFYIx	CxCVIx	CxCVWx	CxCVFx	CxCVCx	CxCVYx	CxCVMx	CxCVVx	CxCVLx	CxCIIx
CxCIWx	CxCIFx	CxCICx	CxCIYx	CxCIMx	CxCIVx	CxCLIx	CxCLWx	CxCLFx	CxCFIx
CxCCIx	CxLVIx	CxLVWx	CxLVFx	CxLVCx	CxLVYx	CxLVMx	CxLVVx	CxLVLx	CxLIIx
CxLIWx	CxLIFx	CxLICx	CxLIYx	CxLIMx	CxLIVx	CxLLIx	CxLLWx	CxLLFx	CxLFIx
CxMVIx	CxMVWx	CxMVFx	CxMVCx	CxMVYx	CxMVMx	CxMVVx	CxMIIx	CxMIWx	CxMIFx
CxMLIx	CxWVIx	CxWVWx	CxWVFx	CxWIIx	CxYVIx	LxIVIx	LxIVWx	LxIVFx	LxIVCx

LxIVYx	LxIVMx	LxIVVx	LxIVLx	LxIVGx	LxIVHx	LxIVTx	LxIVPx	LxIVAx	LxIVNx
LxIVQx	LxIIIx	LxIIWx	LxIIFx	LxIICx	LxIIYx	LxIIMx	LxIIVx	LxIILx	LxIIGx
LxIIHx	LxIITx	LxIIPx	LxIIAx	LxILIx	LxILWx	LxILFx	LxILCx	LxILYx	LxILMx
LxILVx	LxILLx	LxILGx	LxILHx	LxIFIx	LxIFWx	LxIFFx	LxIFCx	LxIFYx	LxIFMx
LxIFVx	LxIFLx	LxIFGx	LxICIx	LxICWx	LxICFx	LxICCx	LxICYx	LxICMx	LxICVx
LxICLx	LxIWIx	LxIWWx	LxIWFx	LxIWCx	LxIWYx	LxIWMx	LxIWVx	LxIWLx	LxIYIx
LxIYWx	LxIYFx	LxIYCx	LxIYYx	LxIYMx	LxIYVx	LxIYLx	LxIMIx	LxIMWx	LxIMFx
LxIMCx	LxIMYx	LxIMMx	LxIMVx	LxIMLx	LxIAIx	LxIAWx	LxIAFx	LxIACx	LxIAYx
LxIAMx	LxIAVx	LxITIx	LxINIx	LxVVIx	LxVVWx	LxVVFx	LxVVCx	LxVVYx	LxVVMx
LxVVVx	LxVVLx	LxVVGx	LxVVHx	LxVVTx	LxVIIx	LxVIWx	LxVIFx	LxVICx	LxVIYx
LxVIMx	LxVIVx	LxVILx	LxVIGx	LxVIHx	LxVLIx	LxVLWx	LxVLFx	LxVLCx	LxVLYx
LxVLMx	LxVLVx	LxVLLx	LxVLGx	LxVFIx	LxVFWx	LxVFFx	LxVFCx	LxVFYx	LxVFMx
LxVFVx	LxVFLx	LxVCIx	LxVCWx	LxVCFx	LxVCCx	LxVCYx	LxVCMx	LxVCVx	LxVCLx
LxVWIx	LxVWWx	LxVWFx	LxVWCx	LxVWYx	LxVWMx	LxVWVx	LxVYIx	LxVYWx	LxVYFx
LxVYCx	LxVYYx	LxVYMx	LxVYVx	LxVMIx	LxVMWx	LxVMFx	LxVMCx	LxVAIx	LxVAWx
LxFVIx	LxFVWx	LxFVFx	LxFVCx	LxFVYx	LxFVMx	LxFVVx	LxFVLx	LxFIIx	LxFIWx
LxFIFx	LxFICx	LxFIYx	LxFIMx	LxFIVx	LxFILx	LxFLIx	LxFLWx	LxFLFx	LxFFIx
LxFCIx	LxFWIx	LxFYIx	LxCVIx	LxCVWx	LxCVFx	LxCVCx	LxCVYx	LxCVMx	LxCVVx
LxCVLx	LxCIIx	LxCIWx	LxCIFx	LxCICx	LxCIYx	LxCIMx	LxCIVx	LxCLIx	LxCLWx
LxCLFx	LxCFIx	LxCCIx	LxLVIx	LxLVWx	LxLVFx	LxLVCx	LxLVYx	LxLVMx	LxLVVx
LxLVLx	LxLIIx	LxLIWx	LxLIFx	LxLICx	LxLIYx	LxLIMx	LxLIVx	LxLLIx	LxLLWx
LxLLFx	LxLFIx	LxMVIx	LxMVWx	LxMVFx	LxMVCx	LxMVYx	LxMVMx	LxMVVx	LxMIIx
LxMIWx	LxMIFx	LxMLIx	LxWVIx	LxWVWx	LxWIIx	LxYVIx	FxIVIx	FxIVWx	FxIVFx
FxIVCx	FxIVYx	FxIVMx	FxIVVx	FxIVLx	FxIVGx	FxIVHx	FxIVTx	FxIVPx	FxIVAx
FxIIIx	FxIIWx	FxIIFx	FxIICx	FxIIYx	FxIIMx	FxIIVx	FxIILx	FxIIGx	FxIIHx
FxIITx	FxILIx	FxILWx	FxILFx	FxILCx	FxILYx	FxILMx	FxILVx	FxILLx	FxILGx
FxIFIx	FxIFWx	FxIFFx	FxIFCx	FxIFYx	FxIFMx	FxIFVx	FxIFLx	FxICIx	FxICWx
FxICFx	FxICCx	FxICYx	FxICMx	FxICVx	FxICLx	FxIWIx	FxIWWx	FxIWFx	FxIWCx
FxIWYx	FxIWMx	FxIWVx	FxIWLx	FxIYIx	FxIYWx	FxIYFx	FxIYCx	FxIYYx	FxIYMx
FxIYVx	FxIYLx	FxIMIx	FxIMWx	FxIMFx	FxIMCx	FxIMYx	FxIMMx	FxIMVx	FxIAIx
FxIAWx	FxIAFx	FxITIx	FxVVIx	FxVVWx	FxVVFx	FxVVCx	FxVVYx	FxVVMx	FxVVVx
FxVVLx	FxVVGx	FxVVHx	FxVIIx	FxVIWx	FxVIFx	FxVICx	FxVIYx	FxVIMx	FxVIVx
FxVILx	FxVIGx	FxVLIx	FxVLWx	FxVLFx	FxVLCx	FxVLYx	FxVLMx	FxVLVx	FxVLLx
FxVFIx	FxVFWx	FxVFFx	FxVFCx	FxVFYx	FxVFMx	FxVFVx	FxVCIx	FxVCWx	FxVCFx
FxVCCx	FxVCYx	FxVCMx	FxVCVx	FxVWIx	FxVWWx	FxVWFx	FxVWCx	FxVWYx	FxVYIx
FxVYWx	FxVYFx	FxVYCx	FxVYYx	FxVMIx	FxVMWx	FxVMFx	FxVAIx	FxFVIx	FxFVWx
FxFVFx	FxFVCx	FxFVYx	FxFVMx	FxFVVx	FxFVLx	FxFIIx	FxFIWx	FxFIFx	FxFICx
FxFIYx	FxFIMx	FxFIVx	FxFLIx	FxFLWx	FxFLFx	FxFFIx	FxCVIx	FxCVWx	FxCVFx
FxCVCx	FxCVYx	FxCVMx	FxCVVx	FxCVLx	FxCIIx	FxCIWx	FxCIFx	FxCICx	FxCLIx
FxCLWx	FxLVIx	FxLVWx	FxLVFx	FxLVCx	FxLVYx	FxLVMx	FxLVVx	FxLIIx	FxLIWx
FxLIFx	FxLLIx	FxMVIx	FxMVWx	FxMVFx	FxMIIx	FxMIWx	FxWVIx	MxIVIx	MxIVWx
MxIVFx	MxIVCx	MxIVYx	MxIVMx	MxIVVx	MxIVLx	MxIVGx	MxIVHx	MxIVTx	MxIIIx
MxIIWx	MxIIFx	MxIICx	MxIIYx	MxIIMx	MxIIVx	MxIILx	MxIIGx	MxIIHx	MxILIx
MxILWx	MxILFx	MxILCx	MxILYx	MxILMx	MxILVx	MxILLx	MxILGx	MxIFIx	MxIFWx
MxIFFx	MxIFCx	MxIFYx	MxIFMx	MxIFVx	MxIFLx	MxICIx	MxICWx	MxICFx	MxICCx
MxICYx	MxICMx	MxICVx	MxICLx	MxIWIx	MxIWWx	MxIWFx	MxIWCx	MxIWYx	MxIWMx
MxIWVx	MxIYIx	MxIYWx	MxIYFx	MxIYCx	MxIYYx	MxIYMx	MxIYVx	MxIMIx	MxIMWx
MxIMFx	MxIAIx	MxIAWx	MxVVIx	MxVVWx	MxVVFx	MxVVCx	MxVVYx	MxVVMx	MxVVVx
MxVVLx	MxVVGx	MxVVHx	MxVIIx	MxVIWx	MxVIFx	MxVICx	MxVIYx	MxVIMx	MxVIVx
MxVILx	MxVIGx	MxVLIx	MxVLWx	MxVLFx	MxVLCx	MxVLYx	MxVLMx	MxVLVx	MxVLLx
MxVFIx	MxVFWx	MxVFFx	MxVFCx	MxVCIx	MxVCWx	MxVCFx	MxVWIx	MxVWWx	MxVWFx
MxVYIx	MxVYWx	MxVYFx	MxVMIx	MxFVIx	MxFVWx	MxFVFx	MxFVCx	MxFVYx	MxFVMx
MxFVVx	MxFIIx	MxFIWx	MxFIFx	MxFLIx	MxCVIx	MxCVWx	MxCVFx	MxCVCx	MxCVYx
MxCIIx	MxCIWx	MxCIFx	MxCLIx	MxLVIx	MxLVWx	MxLVFx	MxLVCx	MxLIIx	MxLIWx
MxLLIx	MxMVIx	MxMVWx	MxMIIx	MxWVIx	WxIVIx	WxIVWx	WxIVFx	WxIVCx	WxIVYx
WxIVMx	WxIVVx	WxIVLx	WxIVGx	WxIVHx	WxIVTx	WxIIIx	WxIIWx	WxIIFx	WxIICx
WxIIYx	WxIIMx	WxIIVx	WxIILx	WxIIGx	WxIIHx	WxILIx	WxILWx	WxILFx	WxILCx
WxILYx	WxILMx	WxILVx	WxILLx	WxILGx	WxIFIx	WxIFWx	WxIFFx	WxIFCx	WxIFYx
WxIFMx	WxIFVx	WxIFLx	WxICIx	WxICWx	WxICFx	WxICCx	WxICYx	WxICMx	WxICVx
WxICLx	WxIWIx	WxIWWx	WxIWFx	WxIWCx	WxIWYx	WxIWMx	WxIWVx	WxIYIx	WxIYWx
WxIYFx	WxIYCx	WxIYYx	WxIYMx	WxIYVx	WxIMIx	WxIMWx	WxIMFx	WxIAIx	WxIAWx
WxVVIx	WxVVWx	WxVVFx	WxVVCx	WxVVYx	WxVVMx	WxVVVx	WxVVLx	WxVVGx	WxVVHx
WxVIIx	WxVIWx	WxVIFx	WxVICx	WxVIYx	WxVIMx	WxVIVx	WxVILx	WxVIGx	WxVLIx
WxVLWx	WxVLFx	WxVLCx	WxVLYx	WxVLMx	WxVLVx	WxVLLx	WxVFIx	WxVFWx	WxVFFx
WxVFCx	WxVCIx	WxVCWx	WxVCFx	WxVWIx	WxVWWx	WxVWFx	WxVYIx	WxVYWx	WxVYFx
WxVMIx	WxFVIx	WxFVWx	WxFVFx	WxFVCx	WxFVYx	WxFVMx	WxFVVx	WxFIIx	WxFIWx
WxFIFx	WxFLIx	WxCVIx	WxCVWx	WxCVFx	WxCVCx	WxCVYx	WxCIIx	WxCIWx	WxCIFx
WxCLIx	WxLVIx	WxLVWx	WxLVFx	WxLVCx	WxLIIx	WxLIWx	WxLLIx	WxMVIx	WxMVWx

WxMIIx	GxIVIx	GxIVWx	GxIVFx	GxIVCx	GxIVYx	GxIVMx	GxIVVx	GxIVLx	GxIVGx
GxIVHx	GxIIIx	GxIIWx	GxIIFx	GxIICx	GxIIYx	GxIIMx	GxIIVx	GxIILx	GxIIGx
GxILIx	GxILWx	GxILFx	GxILCx	GxILYx	GxILMx	GxILVx	GxILLx	GxIFIx	GxIFWx
GxIFFx	GxIFCx	GxICIx	GxICWx	GxICFx	GxIWIx	GxIWWx	GxIWFx	GxIYIx	GxIYWx
GxIYFx	GxIMIx	GxVVIx	GxVVWx	GxVVFx	GxVVCx	GxVVYx	GxVVMx	GxVVVx	GxVVLx
GxVIIx	GxVIWx	GxVIFx	GxVICx	GxVIYx	GxVIMx	GxVIVx	GxVILx	GxVLIx	GxVLWx
GxVLFx	GxVLCx	GxVFIx	GxVCIx	GxVWIx	GxVYIx	GxFVIx	GxFVWx	GxFVFx	GxFIIx
GxCVIx	GxCVWx	GxCIIx	GxLVIx	GxMVIx	YxIVIx	YxIVWx	YxIVFx	YxIVCx	YxIVYx
YxIVMx	YxIVVx	YxIVLx	YxIVGx	YxIVHx	YxIIIx	YxIIWx	YxIIFx	YxIICx	YxIIYx
YxIIMx	YxIIVx	YxIILx	YxIIGx	YxILIx	YxILWx	YxILFx	YxILCx	YxILYx	YxILMx
YxILVx	YxILLx	YxIFIx	YxIFWx	YxIFFx	YxIFCx	YxICIx	YxICWx	YxICFx	YxIWIx
YxIWWx	YxIWFx	YxIYIx	YxIYWx	YxIYFx	YxIMIx	YxVVIx	YxVVWx	YxVVFx	YxVVCx
YxVVYx	YxVVMx	YxVVVx	YxVVLx	YxVIIx	YxVIWx	YxVIFx	YxVICx	YxVIYx	YxVIMx
YxVIVx	YxVILx	YxVLIx	YxVLWx	YxVLFx	YxVLCx	YxVFIx	YxVCIx	YxVWIx	YxVYIx
YxFVIx	YxFVWx	YxFVFx	YxFIIx	YxCVIx	YxCVWx	YxCIIx	YxLVIx	YxMVIx	AxIVIx
AxIVWx	AxIVFx	AxIVCx	AxIVYx	AxIVMx	AxIVVx	AxIVLx	AxIVGx	AxIIIx	AxIIWx
AxIIFx	AxIICx	AxIIYx	AxIIMx	AxIIVx	AxIILx	AxILIx	AxILWx	AxILFx	AxILCx
AxILYx	AxILMx	AxILVx	AxIFIx	AxIFWx	AxIFFx	AxICIx	AxICWx	AxIWIx	AxIYIx
AxIMIx	AxVVIx	AxVVWx	AxVVFx	AxVVCx	AxVVYx	AxVVMx	AxVVVx	AxVVLx	AxVIIx
AxVIWx	AxVIFx	AxVICx	AxVIYx	AxVIMx	AxVIVx	AxVLIx	AxVLWx	AxVLFx	AxVFIx
AxFVIx	AxCVIx	AxLVIx	HxIVIx	HxIVWx	HxIVFx	HxIVCx	HxIVYx	HxIVMx	HxIVVx
HxIVLx	HxIIIx	HxIIWx	HxIIFx	HxIICx	HxIIYx	HxIIMx	HxIIVx	HxILIx	HxILWx
HxILFx	HxIFIx	HxICIx	HxVVIx	HxVVWx	HxVVFx	HxVVCx	HxVVYx	HxVVMx	HxVVVx
HxVIIx	HxVIWx	HxVIFx	HxVLIx	TxIVIx	TxIVWx	TxIVFx	TxIVCx	TxIVYx	TxIVMx
TxIVVx	TxIVLx	TxIIIx	TxIIWx	TxIIFx	TxIICx	TxIIYx	TxIIMx	TxIIVx	TxILIx
TxILWx	TxILFx	TxIFIx	TxVVIx	TxVVWx	TxVVFx	TxVVCx	TxVIIx	TxVIWx	TxVLIx
SxIVIx	SxIVWx	SxIVFx	SxIVCx	SxIVYx	SxIVMx	SxIVVx	SxIIIx	SxIIWx	SxIIFx
SxILIx	SxVVIx	SxVVWx	SxVVFx	SxVIIx	QxIVIx	QxIVWx	QxIVFx	QxIVCx	QxIVYx
QxIVMx	QxIVVx	QxIIIx	QxIIWx	QxIIFx	QxILIx	QxVVIx	QxVVWx	QxVVFx	QxVIIx
KxIVIx	KxIVWx	KxIVFx	KxIVCx	KxIVYx	KxIVMx	KxIVVx	KxIIIx	KxIIWx	KxIIFx
KxILIx	KxVVIx	KxVVWx	KxVIIx	NxIVIx	NxIVWx	NxIVFx	NxIVCx	NxIVYx	NxIIIx
NxIIWx	NxILIx	NxVVIx	NxVVWx	NxVIIx	RxIVIx	RxIVWx	RxIIIx	RxVVIx	DxIVIx
ExIVIx	VxIVxV	VxIVxC	VxIVxI	VxIVxF	VxIVxL	VxIVxY	VxIVxW	VxIVxM	VxIVxA
VxIVxT	VxIVxS	VxIVxH	VxIVxN	VxIVxQ	VxIVxK	VxIVxG	VxIVxR	VxIVxD	VxIIxV
VxIIxC	VxIIxI	VxIIxF	VxIIxL	VxIIxY	VxIIxW	VxIIxM	VxIIxA	VxIIxT	VxIIxS
VxIIxH	VxIIxN	VxIIxQ	VxIIxK	VxILxV	VxILxC	VxILxI	VxILxF	VxILxL	VxILxY
VxILxW	VxILxM	VxILxA	VxILxT	VxILxS	VxILxH	VxIFxV	VxIFxC	VxIFxI	VxIFxF
VxIFxL	VxIFxY	VxIFxW	VxIFxM	VxICxV	VxICxC	VxICxI	VxICxF	VxICxL	VxICxY
VxICxW	VxICxM	VxIWxV	VxIWxC	VxIWxI	VxIWxF	VxIWxL	VxIWxY	VxIWxW	VxIYxV
VxIYxC	VxIYxI	VxIYxF	VxIYxL	VxIYxY	VxIYxW	VxIMxV	VxIMxC	VxIMxI	VxIMxF
VxIMxL	VxIAxV	VxIAxC	VxIAxI	VxITxV	VxVVxV	VxVVxC	VxVVxI	VxVVxF	VxVVxL
VxVVxY	VxVVxW	VxVVxM	VxVVxA	VxVVxT	VxVVxS	VxVVxH	VxVVxN	VxVIxV	VxVIxC
VxVIxI	VxVIxF	VxVIxL	VxVIxY	VxVIxW	VxVIxM	VxVIxA	VxVIxT	VxVIxS	VxVIxH
VxVLxV	VxVLxC	VxVLxI	VxVLxF	VxVLxL	VxVLxY	VxVLxW	VxVLxM	VxVFxV	VxVFxC
VxVFxI	VxVFxF	VxVFxL	VxVCxV	VxVCxC	VxVCxI	VxVCxF	VxVWxV	VxVWxC	VxVWxI
VxVWxF	VxVYxV	VxVYxC	VxVYxI	VxVYxF	VxVMxV	VxVMxC	VxVAxV	VxFVxV	VxFVxC
VxFVxI	VxFVxF	VxFVxL	VxFVxY	VxFVxW	VxFIxV	VxFIxC	VxFIxI	VxFIxF	VxFLxV
VxFLxC	VxFFxV	VxCVxV	VxCVxC	VxCVxI	VxCVxF	VxCVxL	VxCVxY	VxCIxV	VxCIxC
VxCIxI	VxCLxV	VxLVxV	VxLVxC	VxLVxI	VxLVxF	VxLVxL	VxLIxV	VxLIxC	VxLIxI
VxLLxV	VxMVxV	VxMVxC	VxMVxI	VxMIxV	VxWVxV	IxIVxV	IxIVxC	IxIVxI	IxIVxF
IxIVxL	IxIVxY	IxIVxW	IxIVxM	IxIVxA	IxIVxT	IxIVxS	IxIVxH	IxIVxN	IxIVxQ
IxIVxK	IxIVxG	IxIVxR	IxIVxD	IxIIxV	IxIIxC	IxIIxI	IxIIxF	IxIIxL	IxIIxY
IxIIxW	IxIIxM	IxIIxA	IxIIxT	IxIIxS	IxIIxH	IxIIxN	IxILxV	IxILxC	IxILxI
IxILxF	IxILxL	IxILxY	IxILxW	IxILxM	IxILxA	IxILxT	IxILxS	IxILxH	IxIFxV
IxIFxC	IxIFxI	IxIFxF	IxIFxL	IxIFxY	IxIFxW	IxIFxM	IxICxV	IxICxC	IxICxI
IxICxF	IxICxL	IxICxY	IxICxW	IxIWxV	IxIWxC	IxIWxI	IxIWxF	IxIWxL	IxIWxY
IxIYxV	IxIYxC	IxIYxI	IxIYxF	IxIYxL	IxIYxY	IxIMxV	IxIMxC	IxIMxI	IxIMxF
IxIAxV	IxIAxC	IxIAxI	IxVVxV	IxVVxC	IxVVxI	IxVVxF	IxVVxL	IxVVxY	IxVVxW
IxVVxM	IxVVxA	IxVVxT	IxVVxS	IxVVxH	IxVIxV	IxVIxC	IxVIxI	IxVIxF	IxVIxL
IxVIxY	IxVIxW	IxVIxM	IxVIxA	IxVIxT	IxVIxS	IxVIxH	IxVLxV	IxVLxC	IxVLxI
IxVLxF	IxVLxL	IxVLxY	IxVLxW	IxVLxM	IxVFxV	IxVFxC	IxVFxI	IxVFxF	IxVCxV
IxVCxC	IxVCxI	IxVCxF	IxVWxV	IxVWxC	IxVWxI	IxVYxV	IxVYxC	IxVYxI	IxVMxV
IxVMxC	IxVAxV	IxFVxV	IxFVxC	IxFVxI	IxFVxF	IxFVxL	IxFVxY	IxFIxV	IxFIxC
IxFIxI	IxFLxV	IxCVxV	IxCVxC	IxCVxI	IxCVxF	IxCVxL	IxCIxV	IxCIxC	IxCIxI
IxCLxV	IxLVxV	IxLVxC	IxLVxI	IxLVxF	IxLIxV	IxLIxC	IxLIxI	IxLLxV	IxMVxV
IxMVxC	IxMVxI	IxMIxV	IxWVxV	CxIVxV	CxIVxC	CxIVxI	CxIVxF	CxIVxL	CxIVxY
CxIVxW	CxIVxM	CxIVxA	CxIVxT	CxIVxS	CxIVxH	CxIVxN	CxIVxQ	CxIVxK	CxIVxG
CxIIxV	CxIIxC	CxIIxI	CxIIxF	CxIIxL	CxIIxY	CxIIxW	CxIIxM	CxIIxA	CxIIxT

CxIIxS	CxIIxH	CxILxV	CxILxC	CxILxI	CxILxF	CxILxL	CxILxY	CxILxW	CxILxM
CxILxA	CxIFxV	CxIFxC	CxIFxI	CxIFxF	CxIFxL	CxIFxY	CxICxV	CxICxC	CxICxI
CxICxF	CxICxL	CxIWxV	CxIWxC	CxIWxI	CxIWxF	CxIYxV	CxIYxC	CxIYxI	CxIYxF
CxIMxV	CxIMxC	CxIMxI	CxIAxV	CxIAxC	CxVVxV	CxVVxC	CxVVxI	CxVVxF	CxVVxL
CxVVxY	CxVVxW	CxVVxM	CxVVxA	CxVVxT	CxVVxS	CxVVxH	CxVIxV	CxVIxC	CxVIxI
CxVIxF	CxVIxL	CxVIxY	CxVIxW	CxVIxM	CxVLxV	CxVLxC	CxVLxI	CxVLxF	CxVLxL
CxVLxY	CxVFxV	CxVFxC	CxVFxI	CxVCxV	CxVCxC	CxVCxI	CxVWxV	CxVWxC	CxVYxV
CxVYxC	CxVMxV	CxFVxV	CxFVxC	CxFVxI	CxFVxF	CxFIxV	CxFIxC	CxFIxI	CxFLxV
CxCVxV	CxCVxC	CxCVxI	CxCVxF	CxCIxV	CxCIxC	CxCLxV	CxLVxV	CxLVxC	CxLVxI
CxLIxV	CxLIxC	CxMVxV	CxMVxC	CxMIxV	LxIVxV	LxIVxC	LxIVxI	LxIVxF	LxIVxL
LxIVxY	LxIVxW	LxIVxM	LxIVxA	LxIVxT	LxIVxS	LxIVxH	LxIVxN	LxIVxQ	LxIVxK
LxIIxV	LxIIxC	LxIIxI	LxIIxF	LxIIxL	LxIIxY	LxIIxW	LxIIxM	LxIIxA	LxIIxT
LxIIxS	LxIIxH	LxILxV	LxILxC	LxILxI	LxILxF	LxILxL	LxILxY	LxILxW	LxILxM
LxIFxV	LxIFxC	LxIFxI	LxIFxF	LxIFxL	LxIFxY	LxICxV	LxICxC	LxICxI	LxICxF
LxICxL	LxIWxV	LxIWxC	LxIWxI	LxIWxF	LxIYxV	LxIYxC	LxIYxI	LxIYxF	LxIMxV
LxIMxC	LxIMxI	LxIAxV	LxVVxV	LxVVxC	LxVVxI	LxVVxF	LxVVxL	LxVVxY	LxVVxW
LxVVxM	LxVVxA	LxVVxT	LxVVxS	LxVVxH	LxVIxV	LxVIxC	LxVIxI	LxVIxF	LxVIxL
LxVIxY	LxVIxW	LxVIxM	LxVLxV	LxVLxC	LxVLxI	LxVLxF	LxVLxL	LxVLxY	LxVFxV
LxVFxC	LxVFxI	LxVCxV	LxVCxC	LxVWxV	LxVWxC	LxVYxV	LxVYxC	LxVMxV	LxFVxV
LxFVxC	LxFVxI	LxFVxF	LxFIxV	LxFIxC	LxFLxV	LxCVxV	LxCVxC	LxCVxI	LxCIxV
LxCIxC	LxCLxV	LxLVxV	LxLVxC	LxLVxI	LxLIxV	LxMVxV	LxMIxV	FxIVxV	FxIVxC
FxIVxI	FxIVxF	FxIVxL	FxIVxY	FxIVxW	FxIVxM	FxIVxA	FxIVxT	FxIVxS	FxIVxH
FxIIxV	FxIIxC	FxIIxI	FxIIxF	FxIIxL	FxIIxY	FxIIxW	FxIIxM	FxIIxA	FxIIxT
FxIIxS	FxIIxH	FxILxV	FxILxC	FxILxI	FxILxF	FxILxL	FxILxY	FxILxW	FxILxM
FxIFxV	FxIFxC	FxIFxI	FxIFxF	FxICxV	FxICxC	FxICxI	FxICxF	FxIWxV	FxIWxC
FxIWxI	FxIYxV	FxIYxC	FxIYxI	FxIMxV	FxIMxC	FxIAxV	FxVVxV	FxVVxC	FxVVxI
FxVVxF	FxVVxL	FxVVxY	FxVVxW	FxVVxM	FxVVxA	FxVVxT	FxVIxV	FxVIxC	FxVIxI
FxVIxF	FxVIxL	FxVIxY	FxVIxW	FxVIxM	FxVLxV	FxVLxC	FxVLxI	FxVLxF	FxVFxV
FxVFxC	FxVCxV	FxVWxV	FxVYxV	FxFVxV	FxFVxC	FxFVxI	FxFIxV	FxCVxV	FxCVxC
FxCIxV	FxLVxV	FxLVxC	FxLIxV	FxMVxV	MxIVxV	MxIVxC	MxIVxI	MxIVxF	MxIVxL
MxIVxY	MxIVxW	MxIVxM	MxIVxA	MxIVxT	MxIVxS	MxIVxH	MxIIxV	MxIIxC	MxIIxI
MxIIxF	MxIIxL	MxIIxY	MxIIxW	MxIIxM	MxILxV	MxILxC	MxILxI	MxILxF	MxILxL
MxILxY	MxIFxV	MxIFxC	MxIFxI	MxICxV	MxICxC	MxIWxV	MxIWxC	MxIYxV	MxIYxC
MxIMxV	MxVVxV	MxVVxC	MxVVxI	MxVVxF	MxVVxL	MxVVxY	MxVVxW	MxVVxM	MxVIxV
MxVIxC	MxVIxI	MxVIxF	MxVIxL	MxVLxV	MxVLxC	MxVLxI	MxVFxV	MxVCxV	MxFVxV
MxFVxC	MxFIxV	MxCVxV	MxLVxV	WxIVxV	WxIVxC	WxIVxI	WxIVxF	WxIVxL	WxIVxY
WxIVxW	WxIVxM	WxIVxA	WxIVxT	WxIVxS	WxIVxH	WxIIxV	WxIIxC	WxIIxI	WxIIxF
WxIIxL	WxIIxY	WxIIxW	WxIIxM	WxILxV	WxILxC	WxILxI	WxILxF	WxILxL	WxILxY
WxIFxV	WxIFxC	WxIFxI	WxICxV	WxICxC	WxIWxV	WxIWxC	WxIYxV	WxIYxC	WxIMxV
WxVVxV	WxVVxC	WxVVxI	WxVVxF	WxVVxL	WxVVxY	WxVVxW	WxVVxM	WxVIxV	WxVIxC
WxVIxI	WxVIxF	WxVIxL	WxVLxV	WxVLxC	WxVLxI	WxVFxV	WxVCxV	WxFVxV	WxFVxC
WxFIxV	WxCVxV	WxLVxV	GxIVxV	GxIVxC	GxIVxI	GxIVxF	GxIVxL	GxIVxY	GxIVxW
GxIVxM	GxIIxV	GxIIxC	GxIIxI	GxIIxF	GxIIxL	GxILxV	GxILxC	GxILxI	GxIFxV
GxICxV	GxVVxV	GxVVxC	GxVVxI	GxVVxF	GxVIxV	GxVIxC	GxVIxI	GxVLxV	YxIVxV
YxIVxC	YxIVxI	YxIVxF	YxIVxL	YxIVxY	YxIVxW	YxIVxM	YxIIxV	YxIIxC	YxIIxI
YxIIxF	YxIIxL	YxILxV	YxILxC	YxILxI	YxIFxV	YxICxV	YxVVxV	YxVVxC	YxVVxI
YxVVxF	YxVIxV	YxVIxC	YxVIxI	YxVLxV	AxIVxV	AxIVxC	AxIVxI	AxIVxF	AxIVxL
AxIVxY	AxIVxW	AxIIxV	AxIIxC	AxIIxI	AxIIxF	AxILxV	AxILxC	AxVVxV	AxVVxC
AxVVxI	AxVIxV	AxVIxC	AxVLxV	HxIVxV	HxIVxC	HxIVxI	HxIIxV	HxIIxC	HxILxV
HxVVxV	TxIVxV	TxIVxC	TxIVxI	TxIIxV	TxVVxV	SxIVxV	SxIVxC	SxIIxV	QxIVxV
QxIVxC	QxIIxV	KxIVxV	NxIVxV	VxIxIV	VxIxIC	VxIxII	VxIxIF	VxIxIL	VxIxIY
VxIxIW	VxIxIM	VxIxIA	VxIxIT	VxIxIS	VxIxIH	VxIxWV	VxIxWC	VxIxWI	VxIxWF
VxIxWL	VxIxWY	VxIxWW	VxIxWM	VxIxFV	VxIxFC	VxIxFI	VxIxFF	VxIxFL	VxIxFY
VxIxFW	VxIxFM	VxIxCV	VxIxCC	VxIxCI	VxIxCF	VxIxCL	VxIxCY	VxIxYV	VxIxYC
VxIxYI	VxIxYF	VxIxYL	VxIxYY	VxIxMV	VxIxMC	VxIxMI	VxIxMF	VxIxML	VxIxVV
VxIxVC	VxIxVI	VxIxVF	VxIxVL	VxIxLV	VxIxLC	VxIxLI	VxIxLF	VxIxGV	VxVxIV
VxVxIC	VxVxII	VxVxIF	VxVxIL	VxVxIY	VxVxIW	VxVxIM	VxVxWV	VxVxWC	VxVxWI
VxVxWF	VxVxWL	VxVxWY	VxVxFV	VxVxFC	VxVxFI	VxVxFF	VxVxFL	VxVxCV	VxVxCC
VxVxCI	VxVxYV	VxVxYC	VxVxYI	VxVxMV	VxVxMC	VxVxMI	VxVxVV	VxVxVC	VxVxVI
VxVxLV	VxVxLC	VxFxIV	VxFxIC	VxFxWV	VxCxIV	VxCxWV	VxLxIV	IxIxIV	IxIxIC
IxIxII	IxIxIF	IxIxIL	IxIxIY	IxIxIW	IxIxIM	IxIxIA	IxIxIT	IxIxIS	IxIxIH
IxIxWV	IxIxWC	IxIxWI	IxIxWF	IxIxWL	IxIxWY	IxIxWW	IxIxWM	IxIxFV	IxIxFC
IxIxFI	IxIxFF	IxIxFL	IxIxFY	IxIxFW	IxIxFM	IxIxCV	IxIxCC	IxIxCI	IxIxCF
IxIxCL	IxIxCY	IxIxYV	IxIxYC	IxIxYI	IxIxYF	IxIxYL	IxIxMV	IxIxMC	IxIxMI
IxIxMF	IxIxVV	IxIxVC	IxIxVI	IxIxVF	IxIxLV	IxIxLC	IxIxLI	IxIxGV	IxVxIV
IxVxIC	IxVxII	IxVxIF	IxVxIL	IxVxIY	IxVxIW	IxVxIM	IxVxWV	IxVxWC	IxVxWI
IxVxWF	IxVxWL	IxVxFV	IxVxFC	IxVxFI	IxVxFF	IxVxCV	IxVxCC	IxVxCI	IxVxYV
IxVxYC	IxVxYI	IxVxMV	IxVxMC	IxVxVV	IxVxVC	IxVxLV	IxFxIV	IxFxWV	IxCxIV

IxLxIV	CxIxIV	CxIxIC	CxIxII	CxIxIF	CxIxIL	CxIxIY	CxIxIW	CxIxIM	CxIxIA
CxIxWV	CxIxWC	CxIxWI	CxIxWF	CxIxWL	CxIxWY	CxIxWW	CxIxFV	CxIxFC	CxIxFI
CxIxFF	CxIxFL	CxIxFY	CxIxCV	CxIxCC	CxIxCI	CxIxCF	CxIxYV	CxIxYC	CxIxYI
CxIxYF	CxIxMV	CxIxMC	CxIxMI	CxIxVV	CxIxVC	CxIxVI	CxIxLV	CxIxLC	CxIxGV
CxVxIV	CxVxIC	CxVxII	CxVxIF	CxVxIL	CxVxIY	CxVxWV	CxVxWC	CxVxWI	CxVxWF
CxVxFV	CxVxFC	CxVxFI	CxVxCV	CxVxCC	CxVxYV	CxVxMV	CxVxVV	CxVxLV	CxFxIV
CxCxIV	LxIxIV	LxIxIC	LxIxII	LxIxIF	LxIxIL	LxIxIY	LxIxIW	LxIxIM	LxIxWV
LxIxWC	LxIxWI	LxIxWF	LxIxWL	LxIxWY	LxIxWW	LxIxFV	LxIxFC	LxIxFI	LxIxFF
LxIxFL	LxIxFY	LxIxCV	LxIxCC	LxIxCI	LxIxCF	LxIxYV	LxIxYC	LxIxYI	LxIxYF
LxIxMV	LxIxMC	LxIxMI	LxIxVV	LxIxVC	LxIxVI	LxIxLV	LxIxLC	LxVxIV	LxVxIC
LxVxII	LxVxIF	LxVxIL	LxVxIY	LxVxWV	LxVxWC	LxVxWI	LxVxFV	LxVxFC	LxVxFI
LxVxCV	LxVxCC	LxVxYV	LxVxMV	LxVxVV	LxVxLV	LxFxIV	LxCxIV	FxIxIV	FxIxIC
FxIxII	FxIxIF	FxIxIL	FxIxIY	FxIxIW	FxIxIM	FxIxWV	FxIxWC	FxIxWI	FxIxWF
FxIxWL	FxIxFV	FxIxFC	FxIxFI	FxIxFF	FxIxCV	FxIxCC	FxIxCI	FxIxYV	FxIxYC
FxIxYI	FxIxMV	FxIxMC	FxIxVV	FxIxVC	FxIxLV	FxVxIV	FxVxIC	FxVxII	FxVxIF
FxVxWV	FxVxWC	FxVxFV	FxVxFC	FxVxCV	FxVxYV	FxVxMV	FxVxVV	MxIxIV	MxIxIC
MxIxII	MxIxIF	MxIxIL	MxIxIY	MxIxWV	MxIxWC	MxIxWI	MxIxFV	MxIxFC	MxIxFI
MxIxCV	MxIxYV	MxIxMV	MxIxVV	MxIxLV	MxVxIV	MxVxIC	MxVxII	MxVxWV	MxVxFV
WxIxIV	WxIxIC	WxIxII	WxIxIF	WxIxIL	WxIxIY	WxIxWV	WxIxWC	WxIxWI	WxIxFV
WxIxFC	WxIxFI	WxIxCV	WxIxYV	WxIxMV	WxIxVV	WxIxLV	WxVxIV	WxVxIC	WxVxII
WxVxWV	WxVxFV	GxIxIV	GxIxIC	GxIxII	GxIxWV	GxIxFV	GxVxIV	YxIxIV	YxIxIC
YxIxII	YxIxWV	YxIxFV	YxVxIV	AxIxIV	AxIxIC	AxIxWV	AxVxIV	HxIxIV	VxxVIV
VxxVIC	VxxVII	VxxVWV	VxxVFV	VxxIIV	VxxIIC	VxxIWV	IxxVIV	IxxVIC	IxxVII
IxxVWV	IxxVFV	IxxIIV	CxxVIV	CxxVIC	CxxVWV	CxxIIV	LxxVIV	LxxVIC	LxxVWV
LxxIIV	FxxVIV	xIIVIx	xIIVWx	xIIVFx	xIIVCx	xIIVYx	xIIVMx	xIIVVx	xIIVLx
xIIVGx	xIIVHx	xIIIIx	xIIIWx	xIIIFx	xIIICx	xIIIYx	xIIIMx	xIIIVx	xIIILx
xIIIGx	xIIIHx	xIILIx	xIILWx	xIILFx	xIILCx	xIILYx	xIILMx	xIILVx	xIILLx
xIIFIx	xIIFWx	xIIFFx	xIIFCx	xIIFYx	xIIFMx	xIIFVx	xIIFLx	xIICIx	xIICWx
xIICFx	xIICCx	xIICYx	xIICMx	xIICVx	xIIWIx	xIIWWx	xIIWFx	xIIWCx	xIIWYx
xIIWMx	xIIWVx	xIIYIx	xIIYWx	xIIYFx	xIIYCx	xIIYYx	xIIYMx	xIIMIx	xIIMWx
xIIMFx	xIIAIx	xIVVIx	xIVVWx	xIVVFx	xIVVCx	xIVVYx	xIVVMx	xIVVVx	xIVVLx
xIVVGx	xIVIIx	xIVIWx	xIVIFx	xIVICx	xIVIYx	xIVIMx	xIVIVx	xIVILx	xIVLIx
xIVLWx	xIVLFx	xIVLCx	xIVLYx	xIVLMx	xIVLVx	xIVLLx	xIVFIx	xIVFWx	xIVFFx
xIVCIx	xIVCWx	xIVCFx	xIVWIx	xIVWWx	xIVYIx	xIVYWx	xIVMIx	xIFVIx	xIFVWx
xIFVFx	xIFVCx	xIFVYx	xIFIIx	xIFIWx	xIFIFx	xIFLIx	xICVIx	xICVWx	xICVFx
xICVCx	xICIIx	xICIWx	xILVIx	xILVWx	xILVFx	xILIIx	xIMVIx	xIMIIx	xFIVIx
xFIVWx	xFIVFx	xFIVCx	xFIVYx	xFIVMx	xFIVVx	xFIVLx	xFIVGx	xFIVHx	xFIIIx
xFIIWx	xFIIFx	xFIICx	xFIIYx	xFIIMx	xFIIVx	xFIILx	xFIIGx	xFILIx	xFILWx
xFILFx	xFILCx	xFILYx	xFILMx	xFILVx	xFILLx	xFIFIx	xFIFWx	xFIFFx	xFIFCx
xFIFYx	xFICIx	xFICWx	xFICFx	xFIWIx	xFIWWx	xFIWFx	xFIYIx	xFIYWx	xFIYFx
xFIMIx	xFIMWx	xFIAIx	xFVVIx	xFVVWx	xFVVFx	xFVVCx	xFVVYx	xFVVMx	xFVVVx
xFVVLx	xFVVGx	xFVIIx	xFVIWx	xFVIFx	xFVICx	xFVIYx	xFVIMx	xFVIVx	xFVILx
xFVLIx	xFVLWx	xFVLFx	xFVLCx	xFVLYx	xFVFIx	xFVFWx	xFVCIx	xFVWIx	xFVYIx
xFFVIx	xFFVWx	xFFVFx	xFFIIx	xFCVIx	xFCVWx	xFCVFx	xFCIIx	xFLVIx	xFLVWx
xFLIIx	xFMVIx	xYIVIx	xYIVWx	xYIVFx	xYIVCx	xYIVYx	xYIVMx	xYIVVx	xYIVLx
xYIVGx	xYIVHx	xYIIIx	xYIIWx	xYIIFx	xYIICx	xYIIYx	xYIIMx	xYIIVx	xYIILx
xYIIGx	xYILIx	xYILWx	xYILFx	xYILCx	xYILYx	xYILMx	xYILVx	xYILLx	xYIFIx
xYIFWx	xYIFFx	xYIFCx	xYIFYx	xYICIx	xYICWx	xYICFx	xYIWIx	xYIWWx	xYIWFx
xYIYIx	xYIYWx	xYIYFx	xYIMIx	xYIMWx	xYIAIx	xYVVIx	xYVVWx	xYVVFx	xYVVCx
xYVVYx	xYVVMx	xYVVVx	xYVVLx	xYVVGx	xYVIIx	xYVIWx	xYVIFx	xYVICx	xYVIYx
xYVIMx	xYVIVx	xYVILx	xYVLIx	xYVLWx	xYVLFx	xYVLCx	xYVLYx	xYVFIx	xYVFWx
xYVCIx	xYVWIx	xYVYIx	xYFVIx	xYFVWx	xYFVFx	xYFIIx	xYCVIx	xYCVWx	xYCVFx
xYCIIx	xYLVIx	xYLVWx	xYLIIx	xYMVIx	xVIVIx	xVIVWx	xVIVFx	xVIVCx	xVIVYx
xVIVMx	xVIVVx	xVIVLx	xVIVGx	xVIIIx	xVIIWx	xVIIFx	xVIICx	xVIIYx	xVIIMx
xVIIVx	xVIILx	xVILIx	xVILWx	xVILFx	xVILCx	xVILYx	xVILMx	xVILVx	xVILLx
xVIFIx	xVIFWx	xVIFFx	xVICIx	xVICWx	xVICFx	xVIWIx	xVIWWx	xVIYIx	xVIYWx
xVIMIx	xVVVIx	xVVVWx	xVVVFx	xVVVCx	xVVVYx	xVVVMx	xVVVVx	xVVVLx	xVVIIx
xVVIWx	xVVIFx	xVVICx	xVVIYx	xVVIMx	xVVIVx	xVVILx	xVVLIx	xVVLWx	xVVLFx
xVVFIx	xVVCIx	xVFVIx	xVFVWx	xVFIIx	xVCVIx	xVLVIx	xWIVIx	xWIVWx	xWIVFx
xWIVCx	xWIVYx	xWIVMx	xWIVVx	xWIVLx	xWIVGx	xWIIIx	xWIIWx	xWIIFx	xWIICx
xWIIYx	xWIIMx	xWIIVx	xWIILx	xWILIx	xWILWx	xWILFx	xWILCx	xWILYx	xWILMx
xWILVx	xWIFIx	xWIFWx	xWICIx	xWIWIx	xWIYIx	xWVVIx	xWVVWx	xWVVFx	xWVVCx
xWVVYx	xWVVMx	xWVVVx	xWVVLx	xWVIIx	xWVIWx	xWVIFx	xWVICx	xWVIYx	xWVLIx
xWVLWx	xWFVIx	xWCVIx	xLIVIx	xLIVWx	xLIVFx	xLIVCx	xLIVYx	xLIVMx	xLIVVx
xLIVLx	xLIVGx	xLIIIx	xLIIWx	xLIIFx	xLIICx	xLIIYx	xLIIMx	xLIIVx	xLIILx
xLILIx	xLILWx	xLILFx	xLILCx	xLILYx	xLILMx	xLIFIx	xLIFWx	xLICIx	xLIWIx
xLIYIx	xLVVIx	xLVVWx	xLVVFx	xLVVCx	xLVVYx	xLVVMx	xLVVVx	xLVVLx	xLVIIx
xLVIWx	xLVIFx	xLVICx	xLVIYx	xLVLIx	xLVLWx	xLFVIx	xLCVIx	xCIVIx	xCIVWx

xCIVFx	xCIVCx	xCIVYx	xCIVMx	xCIVVx	xCIVLx	xCIIIx	xCIIWx	xCIIFx	xCIICx
xCIIYx	xCIIMx	xCIIVx	xCIILx	xCILIx	xCILWx	xCILFx	xCIFIx	xCICIx	xCVVIx
xCVVWx	xCVVFx	xCVVCx	xCVVYx	xCVVMx	xCVVVx	xCVIIx	xCVIWx	xCVIFx	xCVLIx
xMIVIx	xMIVWx	xMIVFx	xMIVCx	xMIVYx	xMIVMx	xMIVVx	xMIVLx	xMIIIx	xMIIWx
xMIIFx	xMILIx	xMVVIx	xMVVWx	xMVVFx	xMVIIx	xHIVIx	xHIVWx	xHIVFx	xHIVCx
xHIVYx	xHIVMx	xHIVVx	xHIVLx	xHIIIx	xHIIWx	xHIIFx	xHILIx	xHVVIx	xHVVWx
xHVVFx	xHVIIx	xQIVIx	xQIVWx	xQIVFx	xQIVCx	xQIIIx	xQIIWx	xQILIx	xQVVIx
xAIVIx	xAIVWx	xAIVFx	xAIIIx	xAVVIx	xTIVIx	xTIVWx	xTIVFx	xTIIIx	xTVVIx
xNIVIx	xNIVWx	xNIIIx	xNVVIx	xSIVIx	xSIVWx	xSIIIx	xGIVIx	xPIVIx	xIIVxV
xIIVxC	xIIVxI	xIIVxF	xIIVxL	xIIVxY	xIIVxW	xIIVxM	xIIVxA	xIIVxT	xIIVxS
xIIIxV	xIIIxC	xIIIxI	xIIIxF	xIIIxL	xIIIxY	xIIIxW	xIIIxM	xIILxV	xIILxC
xIILxI	xIILxF	xIILxL	xIIFxV	xIIFxC	xIICxV	xIICxC	xIIWxV	xIIYxV	xIIMxV
xIVVxV	xIVVxC	xIVVxI	xIVVxF	xIVVxL	xIVVxY	xIVVxW	xIVIxV	xIVIxC	xIVIxI
xIVIxF	xIVLxV	xIVLxC	xIVFxV	xIFVxV	xICVxV	xILVxV	xFIVxV	xFIVxC	xFIVxI
xFIVxF	xFIVxL	xFIVxY	xFIVxW	xFIVxM	xFIIxV	xFIIxC	xFIIxI	xFIIxF	xFIIxL
xFIIxY	xFILxV	xFILxC	xFILxI	xFIFxV	xFICxV	xFIWxV	xFIYxV	xFVVxV	xFVVxC
xFVVxI	xFVVxF	xFVVxL	xFVIxV	xFVIxC	xFVIxI	xFVLxV	xFFVxV	xYIVxV	xYIVxC
xYIVxI	xYIVxF	xYIVxL	xYIVxY	xYIVxW	xYIVxM	xYIIxV	xYIIxC	xYIIxI	xYIIxF
xYIIxL	xYIIxY	xYILxV	xYILxC	xYILxI	xYIFxV	xYICxV	xYIWxV	xYIYxV	xYVVxV
xYVVxC	xYVVxI	xYVVxF	xYVVxL	xYVIxV	xYVIxC	xYVIxI	xYVLxV	xYFVxV	xVIVxV
xVIVxC	xVIVxI	xVIVxF	xVIVxL	xVIVxY	xVIVxW	xVIIxV	xVIIxC	xVIIxI	xVIIxF
xVILxV	xVILxC	xVIFxV	xVVVxV	xVVVxC	xVVVxI	xVVVxF	xVVIxV	xVVIxC	xVVLxV
xWIVxV	xWIVxC	xWIVxI	xWIVxF	xWIVxL	xWIVxY	xWIIxV	xWIIxC	xWIIxI	xWILxV
xWVVxV	xWVVxC	xWVVxI	xWVIxV	xLIVxV	xLIVxC	xLIVxI	xLIVxF	xLIVxL	xLIIxV
xLIIxC	xLIIxI	xLILxV	xLVVxV	xLVVxC	xLVVxI	xLVIxV	xCIVxV	xCIVxC	xCIVxI
xCIVxF	xCIIxV	xCIIxC	xCILxV	xCVVxV	xCVVxC	xCVIxV	xMIVxV	xMIVxC	xMIIxV
xMVVxV	xHIVxV	xHIVxC	xHIIxV	xHVVxV	xQIVxV	xAIVxV	xIIxIV	xIIxIC	xIIxII
xIIxIF	xIIxIL	xIIxWV	xIIxWC	xIIxWI	xIIxFV	xIIxFC	xIIxCV	xIIxYV	xIIxMV
xIIxVV	xIVxIV	xIVxIC	xIVxWV	xIVxFV	xFIxIV	xFIxIC	xFIxII	xFIxWV	xFIxWC
xFIxFV	xFIxCV	xFVxIV	xYIxIV	xYIxIC	xYIxII	xYIxWV	xYIxWC	xYIxFV	xYIxCV
xYVxIV	xVIxIV	xVIxIC	xVIxWV	xVIxFV	xVVxIV	xWIxIV	xLIxIV	xCIxIV	xxIVIV
xxIVIC	xxIVII	xxIVIF	xxIVIL	xxIVIY	xxIVIW	xxIVIM	xxIVIA	xxIVIT	xxIVIS
xxIVIH	xxIVIN	xxIVIQ	xxIVIK	xxIVIG	xxIVIR	xxIVID	xxIVIE	xxIVWV	xxIVWC
xxIVWI	xxIVWF	xxIVWL	xxIVWY	xxIVWW	xxIVWM	xxIVWA	xxIVWT	xxIVWS	xxIVWH
xxIVWN	xxIVWQ	xxIVWK	xxIVWG	xxIVWR	xxIVFV	xxIVFC	xxIVFI	xxIVFF	xxIVFL
xxIVFY	xxIVFW	xxIVFM	xxIVFA	xxIVFT	xxIVFS	xxIVFH	xxIVFN	xxIVFQ	xxIVFK
xxIVFG	xxIVCV	xxIVCC	xxIVCI	xxIVCF	xxIVCL	xxIVCY	xxIVCW	xxIVCM	xxIVCA
xxIVCT	xxIVCS	xxIVCH	xxIVYV	xxIVYC	xxIVYI	xxIVYF	xxIVYL	xxIVYY	xxIVYW
xxIVYM	xxIVYA	xxIVYT	xxIVYS	xxIVYH	xxIVMV	xxIVMC	xxIVMI	xxIVMF	xxIVML
xxIVMY	xxIVMW	xxIVMM	xxIVMA	xxIVMT	xxIVMS	xxIVMH	xxIVVV	xxIVVC	xxIVVI
xxIVVF	xxIVVL	xxIVVY	xxIVVW	xxIVVM	xxIVVA	xxIVVT	xxIVVS	xxIVVH	xxIVLV
xxIVLC	xxIVLI	xxIVLF	xxIVLL	xxIVLY	xxIVLW	xxIVLM	xxIVLA	xxIVLT	xxIVLS
xxIVLH	xxIVGV	xxIVGC	xxIVGI	xxIVGF	xxIVGL	xxIVGY	xxIVGW	xxIVHV	xxIVHC
xxIVHI	xxIVHF	xxIVTV	xxIVTC	xxIVPV	xxIVPC	xxIVAV	xxIVNV	xxIVQV	xxIIIV
xxIIIC	xxIIII	xxIIIF	xxIIIL	xxIIIY	xxIIIW	xxIIIM	xxIIIA	xxIIIT	xxIIIS
xxIIIH	xxIIIN	xxIIIQ	xxIIIK	xxIIIG	xxIIIR	xxIIWV	xxIIWC	xxIIWI	xxIIWF
xxIIWL	xxIIWY	xxIIWW	xxIIWM	xxIIWA	xxIIWT	xxIIWS	xxIIWH	xxIIWN	xxIIFV
xxIIFC	xxIIFI	xxIIFF	xxIIFL	xxIIFY	xxIIFW	xxIIFM	xxIIFA	xxIIFT	xxIIFS
xxIIFH	xxIICV	xxIICC	xxIICI	xxIICF	xxIICL	xxIICY	xxIICW	xxIICM	xxIICA
xxIICT	xxIICS	xxIICH	xxIIYV	xxIIYC	xxIIYI	xxIIYF	xxIIYL	xxIIYY	xxIIYW
xxIIYM	xxIIYA	xxIIYT	xxIIYS	xxIIMV	xxIIMC	xxIIMI	xxIIMF	xxIIML	xxIIMY
xxIIMW	xxIIMM	xxIIMA	xxIIMT	xxIIVV	xxIIVC	xxIIVI	xxIIVF	xxIIVL	xxIIVY
xxIIVW	xxIIVM	xxIIVA	xxIIVT	xxIILV	xxIILC	xxIILI	xxIILF	xxIILL	xxIILY
xxIILW	xxIILM	xxIIGV	xxIIGC	xxIIGI	xxIIGF	xxIIHV	xxIIHC	xxIIHI	xxIITV
xxIIPV	xxIIAV	xxILIV	xxILIC	xxILII	xxILIF	xxILIL	xxILIY	xxILIW	xxILIM
xxILIA	xxILIT	xxILIS	xxILIH	xxILWV	xxILWC	xxILWI	xxILWF	xxILWL	xxILWY
xxILWW	xxILWM	xxILWA	xxILWT	xxILWS	xxILWH	xxILFV	xxILFC	xxILFI	xxILFF
xxILFL	xxILFY	xxILFW	xxILFM	xxILFA	xxILFT	xxILCV	xxILCC	xxILCI	xxILCF
xxILCL	xxILCY	xxILCW	xxILCM	xxILYV	xxILYC	xxILYI	xxILYF	xxILYL	xxILYY
xxILYW	xxILYM	xxILMV	xxILMC	xxILMI	xxILMF	xxILML	xxILMY	xxILMW	xxILVV
xxILVC	xxILVI	xxILVF	xxILVL	xxILVY	xxILVW	xxILLV	xxILLC	xxILLI	xxILLF
xxILLL	xxILLY	xxILGV	xxILGC	xxILHV	xxIFIV	xxIFIC	xxIFII	xxIFIF	xxIFIL
xxIFIY	xxIFIW	xxIFIM	xxIFIA	xxIFIT	xxIFWV	xxIFWC	xxIFWI	xxIFWF	xxIFWL
xxIFWY	xxIFWW	xxIFWM	xxIFFV	xxIFFC	xxIFFI	xxIFFF	xxIFFL	xxIFFY	xxIFCV
xxIFCC	xxIFCI	xxIFCF	xxIFYV	xxIFYC	xxIFYI	xxIFYF	xxIFMV	xxIFMC	xxIFMI
xxIFMF	xxIFVV	xxIFVC	xxIFVI	xxIFVF	xxIFLV	xxIFLC	xxIFLI	xxIFGV	xxICIV
xxICIC	xxICII	xxICIF	xxICIL	xxICIY	xxICIW	xxICIM	xxICWV	xxICWC	xxICWI
xxICWF	xxICWL	xxICWY	xxICWW	xxICFV	xxICFC	xxICFI	xxICFF	xxICFL	xxICFY

xxICCV	xxICCC	xxICCI	xxICCF	xxICYV	xxICYC	xxICYI	xxICMV	xxICMC	xxICMI
xxICVV	xxICVC	xxICVI	xxICLV	xxICLC	xxIWIV	xxIWIC	xxIWII	xxIWIF	xxIWIL
xxIWIY	xxIWIW	xxIWIM	xxIWWV	xxIWWC	xxIWWI	xxIWWF	xxIWWL	xxIWWY	xxIWFV
xxIWFC	xxIWFI	xxIWFF	xxIWCV	xxIWCC	xxIWCI	xxIWYV	xxIWYC	xxIWYI	xxIWMV
xxIWMC	xxIWMI	xxIWVV	xxIWVC	xxIWVI	xxIWLV	xxIWLC	xxIYIV	xxIYIC	xxIYII
xxIYIF	xxIYIL	xxIYIY	xxIYIW	xxIYIM	xxIYWV	xxIYWC	xxIYWI	xxIYWF	xxIYWL
xxIYWY	xxIYFV	xxIYFC	xxIYFI	xxIYFF	xxIYCV	xxIYCC	xxIYCI	xxIYYV	xxIYYC
xxIYYI	xxIYMV	xxIYMC	xxIYMI	xxIYVV	xxIYVC	xxIYLV	xxIYLC	xxIMIV	xxIMIC
xxIMII	xxIMIF	xxIMIL	xxIMIY	xxIMIW	xxIMWV	xxIMWC	xxIMWI	xxIMWF	xxIMFV
xxIMFC	xxIMFI	xxIMCV	xxIMCC	xxIMYV	xxIMYC	xxIMMV	xxIMVV	xxIMLV	xxIAIV
xxIAIC	xxIAII	xxIAIF	xxIAWV	xxIAWC	xxIAFV	xxIAFC	xxIACV	xxIAYV	xxIAMV
xxIAVV	xxITIV	xxINIV	xxVVIV	xxVVIC	xxVVII	xxVVIF	xxVVIL	xxVVIY	xxVVIW
xxVVIM	xxVVIA	xxVVIT	xxVVIS	xxVVIH	xxVVIN	xxVVIQ	xxVVIK	xxVVIG	xxVVWV
xxVVWC	xxVVWI	xxVVWF	xxVVWL	xxVVWY	xxVVWW	xxVVWM	xxVVWA	xxVVWT	xxVVWS
xxVVWH	xxVVFV	xxVVFC	xxVVFI	xxVVFF	xxVVFL	xxVVFY	xxVVFW	xxVVFM	xxVVFA
xxVVFT	xxVVFS	xxVVFH	xxVVCV	xxVVCC	xxVVCI	xxVVCF	xxVVCL	xxVVCY	xxVVCW
xxVVCM	xxVVCA	xxVVCT	xxVVCS	xxVVYV	xxVVYC	xxVVYI	xxVVYF	xxVVYL	xxVVYY
xxVVYW	xxVVYM	xxVVYA	xxVVMV	xxVVMC	xxVVMI	xxVVMF	xxVVML	xxVVMY	xxVVMW
xxVVMM	xxVVVV	xxVVVC	xxVVVI	xxVVVF	xxVVVL	xxVVVY	xxVVVW	xxVVVM	xxVVLV
xxVVLC	xxVVLI	xxVVLF	xxVVLL	xxVVLY	xxVVLW	xxVVLM	xxVVGV	xxVVGC	xxVVGI
xxVVGF	xxVVHV	xxVVHC	xxVVTV	xxVIIV	xxVIIC	xxVIII	xxVIIF	xxVIIL	xxVIIY
xxVIIW	xxVIIM	xxVIIA	xxVIIT	xxVIIS	xxVIIH	xxVIWV	xxVIWC	xxVIWI	xxVIWF
xxVIWL	xxVIWY	xxVIWW	xxVIWM	xxVIWA	xxVIWT	xxVIWS	xxVIFV	xxVIFC	xxVIFI
xxVIFF	xxVIFL	xxVIFY	xxVIFW	xxVIFM	xxVIFA	xxVICV	xxVICC	xxVICI	xxVICF
xxVICL	xxVICY	xxVICW	xxVICM	xxVIYV	xxVIYC	xxVIYI	xxVIYF	xxVIYL	xxVIYY
xxVIYW	xxVIMV	xxVIMC	xxVIMI	xxVIMF	xxVIML	xxVIMY	xxVIMW	xxVIVV	xxVIVC
xxVIVI	xxVIVF	xxVIVL	xxVIVY	xxVIVW	xxVILV	xxVILC	xxVILI	xxVILF	xxVILL
xxVIGV	xxVIGC	xxVIHV	xxVLIV	xxVLIC	xxVLII	xxVLIF	xxVLIL	xxVLIY	xxVLIW
xxVLIM	xxVLIA	xxVLIT	xxVLIS	xxVLWV	xxVLWC	xxVLWI	xxVLWF	xxVLWL	xxVLWY
xxVLWW	xxVLWM	xxVLFV	xxVLFC	xxVLFI	xxVLFF	xxVLFL	xxVLFY	xxVLFW	xxVLCV
xxVLCC	xxVLCI	xxVLCF	xxVLYV	xxVLYC	xxVLYI	xxVLYF	xxVLMV	xxVLMC	xxVLMI
xxVLMF	xxVLVV	xxVLVC	xxVLVI	xxVLVF	xxVLLV	xxVLLC	xxVLLI	xxVLGV	xxVFIV
xxVFIC	xxVFII	xxVFIF	xxVFIL	xxVFIY	xxVFIW	xxVFWV	xxVFWC	xxVFWI	xxVFWF
xxVFFV	xxVFFC	xxVFFI	xxVFCV	xxVFCC	xxVFYV	xxVFYC	xxVFMV	xxVFVV	xxVFLV
xxVCIV	xxVCIC	xxVCII	xxVCIF	xxVCIL	xxVCIY	xxVCWV	xxVCWC	xxVCWI	xxVCFV
xxVCFC	xxVCFI	xxVCCV	xxVCYV	xxVCMV	xxVCVV	xxVCLV	xxVWIV	xxVWIC	xxVWII
xxVWIF	xxVWIL	xxVWWV	xxVWWC	xxVWWI	xxVWFV	xxVWFC	xxVWCV	xxVWYV	xxVWMV
xxVWVV	xxVYIV	xxVYIC	xxVYII	xxVYIF	xxVYIL	xxVYWV	xxVYWC	xxVYWI	xxVYFV
xxVYFC	xxVYCV	xxVYYV	xxVYMV	xxVYVV	xxVMIV	xxVMIC	xxVMII	xxVMWV	xxVMWC
xxVMFV	xxVMCV	xxVAIV	xxVAIC	xxVAWV	xxFVIV	xxFVIC	xxFVII	xxFVIF	xxFVIL
xxFVIY	xxFVIW	xxFVIM	xxFVWV	xxFVWC	xxFVWI	xxFVWF	xxFVWL	xxFVWY	xxFVFV
xxFVFC	xxFVFI	xxFVFF	xxFVCV	xxFVCC	xxFVCI	xxFVYV	xxFVYC	xxFVYI	xxFVMV
xxFVMC	xxFVVV	xxFVVC	xxFVLV	xxFIIV	xxFIIC	xxFIII	xxFIIF	xxFIIL	xxFIIY
xxFIWV	xxFIWC	xxFIWI	xxFIFV	xxFIFC	xxFIFI	xxFICV	xxFIYV	xxFIMV	xxFIVV
xxFILV	xxFLIV	xxFLIC	xxFLII	xxFLWV	xxFLFV	xxFFIV	xxFCIV	xxFWIV	xxCVIV
xxCVIC	xxCVII	xxCVIF	xxCVIL	xxCVIY	xxCVIW	xxCVWV	xxCVWC	xxCVWI	xxCVWF
xxCVFV	xxCVFC	xxCVFI	xxCVFF	xxCVCV	xxCVCC	xxCVYV	xxCVYC	xxCVMV	xxCVMC
xxCVVV	xxCVVC	xxCVLV	xxCIIV	xxCIIC	xxCIII	xxCIIF	xxCIWV	xxCIWC	xxCIWI
xxCIFV	xxCIFC	xxCICV	xxCIYV	xxCIMV	xxCIVV	xxCLIV	xxCLIC	xxCLWV	xxCLFV
xxCFIV	xxLVIV	xxLVIC	xxLVII	xxLVIF	xxLVIL	xxLVIY	xxLVIW	xxLVWV	xxLVWC
xxLVWI	xxLVWF	xxLVFV	xxLVFC	xxLVFI	xxLVCV	xxLVCC	xxLVYV	xxLVYC	xxLVMV
xxLVVV	xxLVLV	xxLIIV	xxLIIC	xxLIII	xxLIIF	xxLIWV	xxLIWC	xxLIFV	xxLIFC
xxLICV	xxLIYV	xxLIMV	xxLIVV	xxLLIV	xxLLIC	xxLLWV	xxLLFV	xxLFIV	xxMVIV
xxMVIC	xxMVII	xxMVIF	xxMVWV	xxMVWC	xxMVFV	xxMVFC	xxMVCV	xxMVYV	xxMVMV
xxMVVV	xxMIIV	xxMIIC	xxMIWV	xxMIFV	xxMLIV	xxWVIV	xxWVIC	xxWVWV	xxWIIV
xxYVIV									

Table S2: Amyloid patterns for restricted substitutions with small, non-polar residues

In the table we list all the existing minimal patterns of amyloid-forming hexapeptides, computed from the SVM model of the Budapest Amyloid Predictor, when the substitutions for the positions, denoted by "x" are allowed by small, non-polar residues, namely G, A, S and T. The table contains 411 patterns.

0	1	2	3	4	5	6	7	8	9
VIIxxx	IIIxxx	VxIVxx	VxIIxx	VxILxx	VxIFxx	VxICxx	VxIWxx	VxIYxx	VxIMxx
VxVVxx	VxVIxx	VxVLxx	VxVFxx	VxFVxx	VxCVxx	VxLVxx	IxIVxx	IxIIxx	IxILxx
IxIFxx	IxICxx	IxIWxx	IxIYxx	IxVVxx	IxVIxx	IxVLxx	IxVFxx	IxFVxx	IxCVxx
IxLVxx	CxIVxx	CxIIxx	CxILxx	CxIFxx	CxICxx	CxIWxx	CxIYxx	CxVVxx	CxVIxx
CxVLxx	CxFVxx	LxIVxx	LxIIxx	LxILxx	LxIFxx	LxICxx	LxIWxx	LxVVxx	LxVIxx
LxVLxx	FxIVxx	FxIIxx	FxILxx	FxVVxx	FxVIxx	FxVLxx	MxIVxx	MxIIxx	MxILxx
MxVVxx	MxVIxx	WxIVxx	WxIIxx	WxILxx	WxVVxx	WxVIxx	GxIVxx	GxIIxx	GxVVxx
YxIVxx	YxIIxx	YxVVxx	AxIVxx	VxIxIx	VxIxWx	VxIxFx	VxIxCx	VxIxYx	VxIxMx
VxIxVx	VxIxLx	VxVxIx	VxVxWx	VxVxFx	IxIxIx	IxIxWx	IxIxFx	IxIxCx	IxIxYx
IxIxMx	IxIxVx	IxVxIx	IxVxWx	IxVxFx	CxIxIx	CxIxWx	CxIxFx	CxIxCx	CxIxYx
CxVxIx	LxIxIx	LxIxWx	LxIxFx	LxIxCx	LxVxIx	FxIxIx	FxIxWx	FxIxFx	FxVxIx
MxIxIx	WxIxIx	VxIxxV	VxIxxC	VxVxxV	IxIxxV	IxIxxC	CxIxxV	LxIxxV	xIIVxx
xIIIxx	xIILxx	xIIFxx	xIICxx	xIIWxx	xIIYxx	xIVVxx	xIVIxx	xIVLxx	xIFVxx
xFIVxx	xFIIxx	xFILxx	xFIFxx	xFVVxx	xFVIxx	xFVLxx	xYIVxx	xYIIxx	xYILxx
xYIFxx	xYVVxx	xYVIxx	xYVLxx	xVIVxx	xVIIxx	xVILxx	xVVVxx	xVVIxx	xWIVxx
xWIIxx	xWILxx	xWVVxx	xWVIxx	xLIVxx	xLIIxx	xLILxx	xLVVxx	xLVIxx	xCIVxx
xCIIxx	xCVVxx	xMIVxx	xHIVxx	xIIxIx	xIIxWx	xIIxFx	xIIxCx	xIIxYx	xIVxIx
xIVxWx	xFIxIx	xFIxWx	xFIxFx	xFVxIx	xYIxIx	xYIxWx	xYIxFx	xYVxIx	xVIxIx
xVIxWx	xWIxIx	xLIxIx	xIIxxV	xFIxxV	xYIxxV	xxIVIx	xxIVWx	xxIVFx	xxIVCx
xxIVYx	xxIVMx	xxIVVx	xxIVLx	xxIVGx	xxIVHx	xxIVTx	xxIIIx	xxIIWx	xxIIFx
xxIICx	xxIIYx	xxIIMx	xxIIVx	xxIILx	xxIIGx	xxIIHx	xxILIx	xxILWx	xxILFx
xxILCx	xxILYx	xxILMx	xxILVx	xxILLx	xxILGx	xxIFIx	xxIFWx	xxIFFx	xxIFCx
xxIFYx	xxIFMx	xxIFVx	xxIFLx	xxICIx	xxICWx	xxICFx	xxICCx	xxICYx	xxICMx
xxICVx	xxICLx	xxIWIx	xxIWWx	xxIWFx	xxIWCx	xxIWYx	xxIWMx	xxIWVx	xxIYIx
xxIYWx	xxIYFx	xxIYCx	xxIYYx	xxIYMx	xxIYVx	xxIMIx	xxIMWx	xxIMFx	xxIAIx
xxIAWx	xxVVIx	xxVVWx	xxVVFx	xxVVCx	xxVVYx	xxVVMx	xxVVVx	xxVVLx	xxVVGx
xxVVHx	xxVIIx	xxVIWx	xxVIFx	xxVICx	xxVIYx	xxVIMx	xxVIVx	xxVILx	xxVIGx
xxVLIx	xxVLWx	xxVLFx	xxVLCx	xxVLYx	xxVLMx	xxVLVx	xxVLLx	xxVFIx	xxVFWx
xxVFFx	xxVFCx	xxVCIx	xxVCWx	xxVCFx	xxVWIx	xxVWWx	xxVWFx	xxVYIx	xxVYWx
xxVYFx	xxVMIx	xxFVIx	xxFVWx	xxFVFx	xxFVCx	xxFVYx	xxFVMx	xxFVVx	xxFIIx
xxFIWx	xxFIFx	xxFLIx	xxCVIx	xxCVWx	xxCVFx	xxCVCx	xxCVYx	xxCIIx	xxCIWx
xxCIFx	xxCLIx	xxLVIx	xxLVWx	xxLVFx	xxLVCx	xxLIIx	xxLIWx	xxLLIx	xxMVIx
xxMVWx	xxMIIx	xxWVIx	xxIVxV	xxIVxC	xxIVxI	xxIVxF	xxIVxL	xxIVxY	xxIVxW
xxIVxM	xxIVxA	xxIVxT	xxIVxS	xxIVxH	xxIIxV	xxIIxC	xxIIxI	xxIIxF	xxIIxL
xxIIxY	xxIIxW	xxIIxM	xxILxV	xxILxC	xxILxI	xxILxF	xxILxL	xxILxY	xxIFxV
xxIFxC	xxIFxI	xxICxV	xxICxC	xxIWxV	xxIWxC	xxIYxV	xxIYxC	xxIMxV	xxVVxV
xxVVxC	xxVVxI	xxVVxF	xxVVxL	xxVVxY	xxVVxW	xxVVxM	xxVIxV	xxVIxC	xxVIxI
xxVIxF	xxVIxL	xxVIxY	xxVLxV	xxVLxC	xxVLxI	xxVFxV	xxVCxV	xxFVxV	xxFVxC
xxFIxV	xxCVxV	xxLVxV	xxIxIV	xxIxIC	xxIxII	xxIxIF	xxIxIL	xxIxIY	xxIxIW
xxIxWV	xxIxWC	xxIxWI	xxIxWF	xxIxFV	xxIxFC	xxIxFI	xxIxCV	xxIxCC	xxIxYV
xxIxYC	xxIxMV	xxIxVV	xxIxLV	xxVxIV	xxVxIC	xxVxII	xxVxWV	xxVxWC	xxVxFV
xxVxCV									
